# Baseline techno-economic and life cycle assessment of black mass production

**DOI:** 10.1016/j.isci.2026.117729

**Published:** 2026-10-01

**Authors:** Tanongsak Yingnakorn, Chatisa Kansomket, Chaiyasit Longbutsri, Sean Scott, Tapany Patcharawit, Jake M. Yang, Andrew P. Abbott, Sakhob Khumkoa

**Affiliations:** 1 School of Metallurgical Engineering, Suranaree University of Technology, Nakhon Ratchasima 30000, Thailand; 2 School of Chemistry, University of Leicester, Leicester LE1 7RH, UK

**Keywords:** life cycle assessment, black mass, techno-economic assessment, lithium-ion batteries, electric vehicle, mechanical separation, baseline, carbon footprint

## Abstract

Recycling lithium-ion batteries hinges on economically producing black mass—the electrode powder concentrate that feeds downstream metal recovery—yet public baselines pairing real process economics with environmental impacts remain scarce. Here we show that a purely mechanical flowsheet—shredding, zigzag air classification, and circular screen vibration—applied identically to two structurally distinct feedstocks recovers black mass at 93.28 wt % separation efficiency from electric-vehicle modules and 75.77 wt % from mobile phone cells. Built on measured pilot data, loaded processing costs of $0.31–0.73/kg yield net profits of $4.39–5.53/kg under realistic feedstock purchase prices, with a probability of unprofitability below 0.2% across simulated market scenarios. Life cycle assessment attributes most impacts to grid electricity, with a global warming potential of 0.210–0.241 kg CO_2_ eq./kg input; a low-carbon grid cuts this by 62.8%. This paired baseline enables benchmarking of emerging recycling routes and supports circular battery supply chains.

## Highlights

• Mechanical flowsheet recovers black mass at up to 93.28 wt % separation efficiency
• Loaded processing cost of $0.31–0.73/kg with net profit of $4.39–5.53/kg
• Process stays profitable in over 99.8% of simulated market price scenarios
• Low-carbon grids cut global warming potential of black mass output by 62.8%

## Graphical abstract

**Figure:**
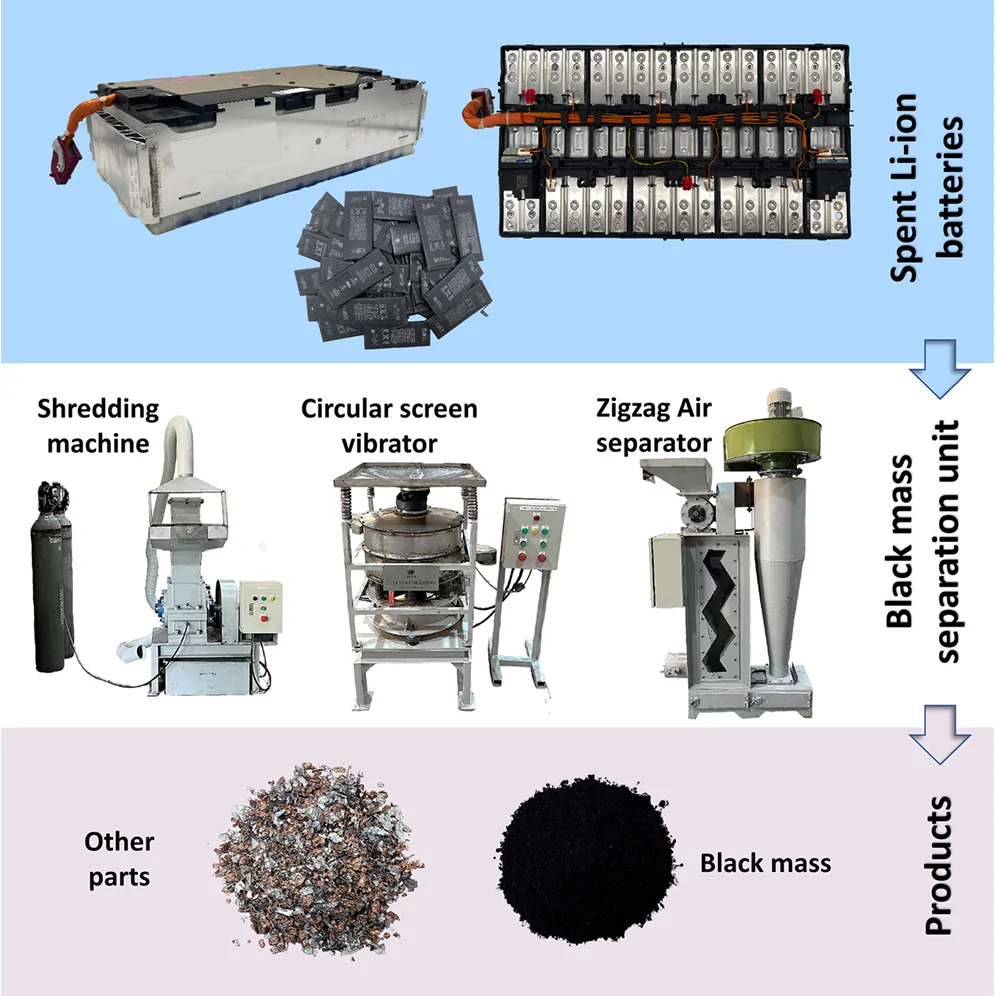

## Teaser

Environmental science; Applied sciences

## Introduction

The electrification of transportation is one of the major contributors to addressing climate change.^1^^,^^2^^,^^3^ Lithium-ion batteries (LIB) are seen as a valuable tool in road electrification and in electricity storage. However, the rapid expansion of LIB deployment poses significant life cycle environmental impacts and critical resource supply risks.^4^^,^^5^^,^^6^^,^^7^ Consequently, electrification is only fully sustainable if energy is derived from renewable sources and battery materials are managed within a true circular economy. Most lithium-ion batteries use relatively rare materials such as cobalt, nickel, and lithium, so it is essential to create efficient recycling processes to recover high-purity materials in high yields.^4^ From a life cycle analysis (LCA) perspective, physical methods are preferable with minimal energy and chemical inputs.^5^^,^^8^ Recycling methods can be split into 3 categories: pyrometallurgical, hydrometallurgical, and physical.^9^ When devising recycling flowsheets, it is imperative to construct them using techno-economic and life cycle analysis to ensure the maximum impact; however, there is very little data available in the literature using this approach.^10^^,^^11^

Legacy methods such as pyro- and hydro-metallurgy have been deployed to recycle lithium-ion battery wastes at scale; this approach has been reviewed comprehensively elsewhere in the literature.^12^^,^^13^^,^^14^^,^^15^^,^^16^ The downside of these processes is the relatively long-looped recycling procedures, and the processes themself are neither cheap nor environmentally benign, as it takes the battery materials all the way back to its battery precursors, which are worth less. The average price for pristine NMC 811 is $50–120 per kg,^17^^,^^18^^,^^19^ and the total cost for the precursor materials required to synthesize 1 kg of NMC 811 is $19–23/kg.^20^ For instance, Sony Electronics has smelted spent LIBs at approximately 1000 °C to evaporate organic compounds, lithium, and fluoride.^2^ The resulting residual slag then undergoes hydrometallurgical treatment to obtain pure cobalt, although lithium recovery remains challenging due to its escape as flue gas.^21^ Alternatively, direct recycling is a much more effective method that avoids the destruction of the original battery crystal structure, allowing direct recovery of end-of-life (EoL) anodes and cathodes for reuse in new batteries. Studies have shown that regenerated lithium cobalt oxide (LCO), nickel manganese cobalt oxide (NMC), and lithium iron phosphate (LFP) materials produced via direct recycling can be up to 43%, 16–27%, and 15% less expensive than their virgin counterparts, respectively.^22^^,^^23^

The presence of residual electrical energy in spent LIBs poses substantial safety risks during the disassembly and shredding processes. To mitigate these hazards, it is essential to discharge the batteries prior to these operations. There are different ways to discharge batteries.^24^ Electrical discharge involves connecting the battery to a resistor to dissipate energy.^25^^,^^26^ Solution discharge utilizes brine or salt solutions, such as NaCl, Na_2_SO_4_, FeSO_4_, or MnSO_4_, to chemically neutralize the battery, but this can lead to corrosion of the current collector foils.^27^^,^^28^ Cryogenic pre-treatment (about −175 to −200 °C) involves immersing the battery in liquid nitrogen to solidify the electrolyte and any metallic lithium, thereby reducing the risk of thermal runaway.^29^^,^^30^ In-process stabilization techniques, such as comminution under a water spray or an inert gas, can also help to control potential hazards during the recycling process.^24^^,^^30^ After batteries are fully discharged, efficient separation processes are crucial in LIB recycling to minimize the loss of valuable materials and to reduce the overall operation cost.^24^ The initial step typically involves comminution, using crushing devices to reduce the battery’s size. Subsequent mechanical separation techniques, such as size separation, magnetic separation, and density separation, are employed to produce higher-purity material streams.^31^^,^^32^^,^^33^^,^^34^ The physical separation process produces various components, including plastic, separator, casing, tabs, current conductor, and a “*black mass*”*.* This black mass, containing valuable anode and cathode active materials, is the most valued material in batteries and requires further processing steps such as the legacy pyro- and hydro-metallurgical methods or direct methods.^35^^,^^36^

While the literature acknowledges the critical step of black mass separation from current collectors in lithium-ion battery recycling,^37^^,^^38^^,^^39^ comprehensive studies detailing semi-pilot-scale operations, comparative machine assessments, and the influence of diverse end-of-life battery sources remain limited. This research addresses this gap by investigating black mass separation’s technical feasibility and efficiency, aiming for industrial scalability, which is essential for subsequent valuable metal recovery. Employing two distinct battery sources, an electric vehicle (EV) module and mobile phone cells (representing small electronic devices), the study evaluates the separation process. The EV modules and mobile phone battery cells underwent discharge before direct processing via two distinct separation pathways. A comprehensive cost-benefit analysis, integrating economic and environmental considerations, was conducted to provide a benchmark against which other processes could be compared. The overarching objective was to establish a cost-effective and environmentally sustainable black mass separation methodology, ensuring the economic justification of the separated material’s value. A key methodological challenge addressed by this work is the need for a cost-effective, sustainable pre-treatment to maximize yield for downstream recycling. While acknowledging the inherent limitation that mechanical black mass separation cannot efficiently recover individual elements or directly resynthesize cathode material, the novelty of the proposed mechanical approach lies in its capacity to deliver a high-grade cathodic concentrate in black mass with high recovery yield. This robust physical and mechanical separation is paramount for maximizing the efficiency and cost-effectiveness of mainstream downstream recycling routes (e.g., pyrometallurgy or hydrometallurgy) by supplying a highly concentrated feedstock.

## Results and discussion

This study utilized a spent electric vehicle battery module and mobile phone batteries as input for baseline techno-economic and life cycle assessment of black mass production. The batteries underwent a controlled discharge process using distinct methodologies. For the electric vehicle battery module, a custom-designed discharger is employed to discharge the module from about 28 V to a voltage below 0.1 V. In contrast, the small device (mobile phone) batteries are discharged by immersing them in a 10 wt % NaCl solution after removal of the protective casing due to the constraints of discharging individual batteries.^40^^,^^41^ This battery stabilization process was intended to prevent short circuits and the consequent rapid escalation of internal battery temperature and, therefore, fire and explosion hazards. An ideal state of charge (SOC) for safe handling is below 2%, with an optimal level of 0%, corresponding to a voltage below 2.0 V.^42^^,^^43^ After the discharge phase, the two types of batteries are disassembled manually, EV and mobile phone, examined, weighed, and their internal components categorized so as to obtain a baseline (Figures 1C and 1D) for the subsequent optimization of shredding and black-mass separation procedures using the custom-built separation unit (Figure 2).

**Figure 1:**
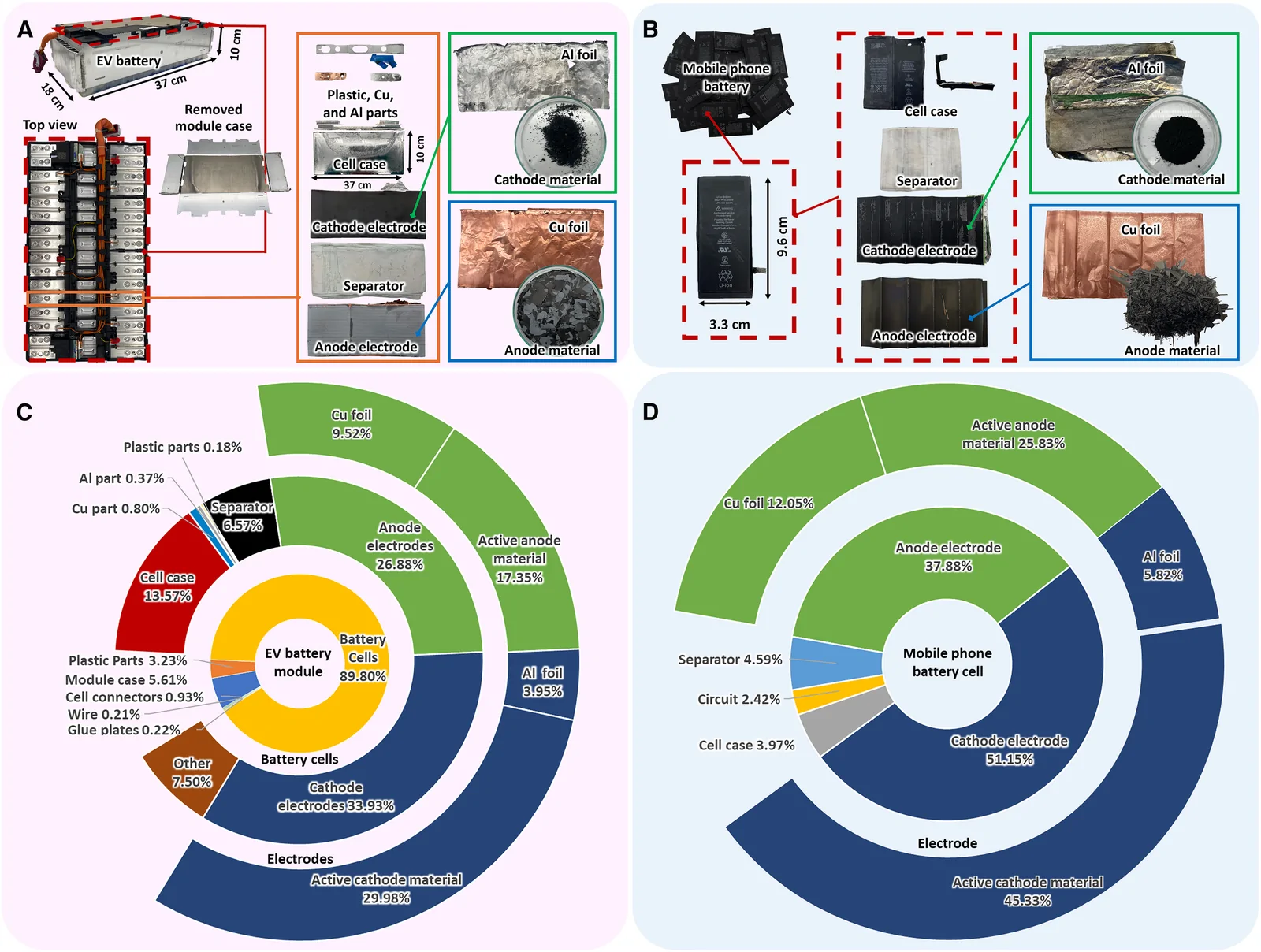
Battery feedstocks and their component mass distributions Components of (A) the electric vehicle and (B) mobile phone batteries used in this study (see the full version of disassembled components in Figure S4). Images (C) and (D) show diagrams of the weight percentage average of an EV battery module and a mobile phone battery cell, respectively. Values in (C) and (D) are mean wt %; *n* = 3 cells (EV) and *n* = 3 cells (MP).

**Figure 2:**
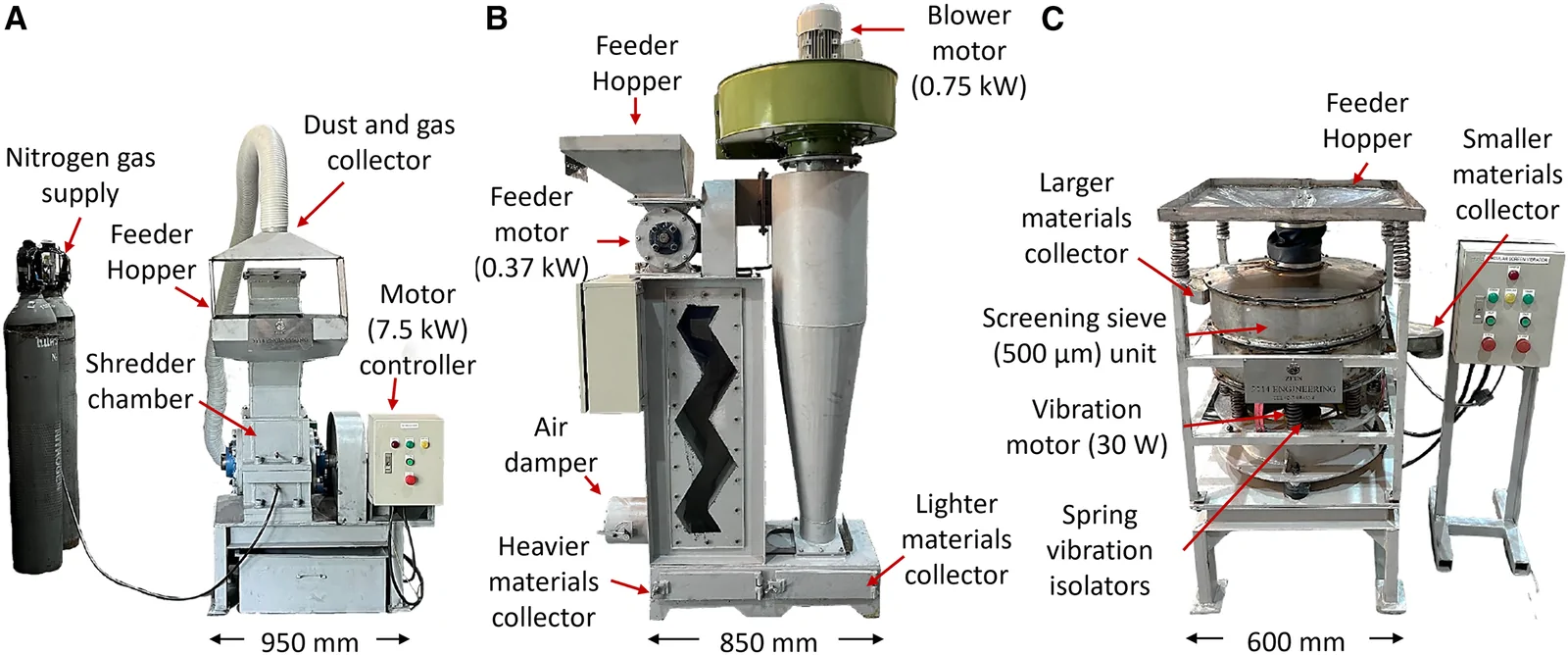
Mechanical separation unit Separation unit consisting of (A) shredder, (B) zigzag air separator, and (C) circular screen vibrator machines.

The scalability of spent battery recycling requires automated mechanical separation to establish the economic and safety foundation. The subsequent analysis integrates these foundational findings, beginning with the detailed characterization of electrode materials. Discussion proceeds to the pivotal mechanical shredding and separation results for both EV and mobile phone cells, which demonstrate the essential role of tailored, high-throughput mechanical strategies. Critically, this mechanical approach provides the necessary feedstock quality—a direct determinant for the cost-efficiency of downstream processing and profitability. The section concludes by quantifying viability and sustainability through techno-economic and life cycle analyses.

### Characterization of electrode materials

The characterization of electrode materials from spent EV and MP batteries was conducted via SEM to elucidate their respective material compositions. Figure 3 presents the surface morphologies of the cathode and anode electrodes. In EV batteries (Figure 3A), the anode structure revealed a central copper foil layer measuring 11.38 (±0.34) μm, sandwiched between graphite layers of 77.52 (±3.23) μm and 37.07 (±2.66) μm. Notably, EDS analysis identified an Al_2_O_3_ layer on the anode surface (see SEM-EDS details in Figure S5), potentially indicative of a coating applied to mitigate irreversible lithium metal deposition and consequent capacity degradation during initial cycling.^44^^,^^45^^,^^46^ The EV cathode cross-section exhibited a 14.76 (±1.21) μm aluminum foil core, coated with approximately 44 μm and 53 μm of nickel-manganese-cobalt (NMC) active material. EDS of the EV active cathode material yielded a mean chemical composition of 20.38 wt % Ni, 8.05 wt % Mn, and 10.22 wt % Co. This elemental quantification approximates a Ni:Mn:Co atomic ratio of 0.52:0.22:0.26. This ratio is close to the established Ni-rich NMC (LiNi_x_Mn_y_Co_1-x-y_O_2_, where x ≥ 0.5) cathode materials, such as LiNi_0.5_Mn_0.3_Co_0.2_O_2_ (NMC532), LiNi_0.6_Mn_0.2_Co_0.2_O_2_ (NMC622), and is within the variance often observed for these types of materials.^47^^,^^48^ X-ray diffraction (XRD) patterns of the EV cathode materials, shown in Figure S6, support the presence of NMC622. Although X-ray diffraction (XRD) patterns matched the characteristic reflections of NMC622, unambiguous phase identification based solely on XRD analysis is challenging due to the high degree of structural similarity across various NMC compositions.^49^^,^^50^^,^^51^ In contrast, the mobile phone battery electrode analysis (Figure 3B) revealed substantially thinner current collector foils, measuring approximately 4.34 μm for the anode and 6.85 μm for the cathode. EDS analysis of the mobile phone battery cathode indicated a composition of 33.94 wt % Co and 22.95 wt % O, consistent with a LiCoO_2_ active material. This identification was further corroborated by XRD analysis (Figure S3), which confirmed the presence of graphite on the anode surface and LiCoO_2_ on the cathode surface. Both EV and MP electrodes exhibited trace amounts of fluorine (F) and phosphorus (P), likely residual components from the dried electrolyte, which commonly utilizes lithium hexafluorophosphate (LiPF_6_).^52^^,^^53^ Furthermore, the detection of F and carbon (C) may indicate the presence of polyvinylidene fluoride (PVDF), a widely utilized binder material in lithium-ion battery electrodes.^54^^,^^55^^,^^56^^,^^57^

**Figure 3:**
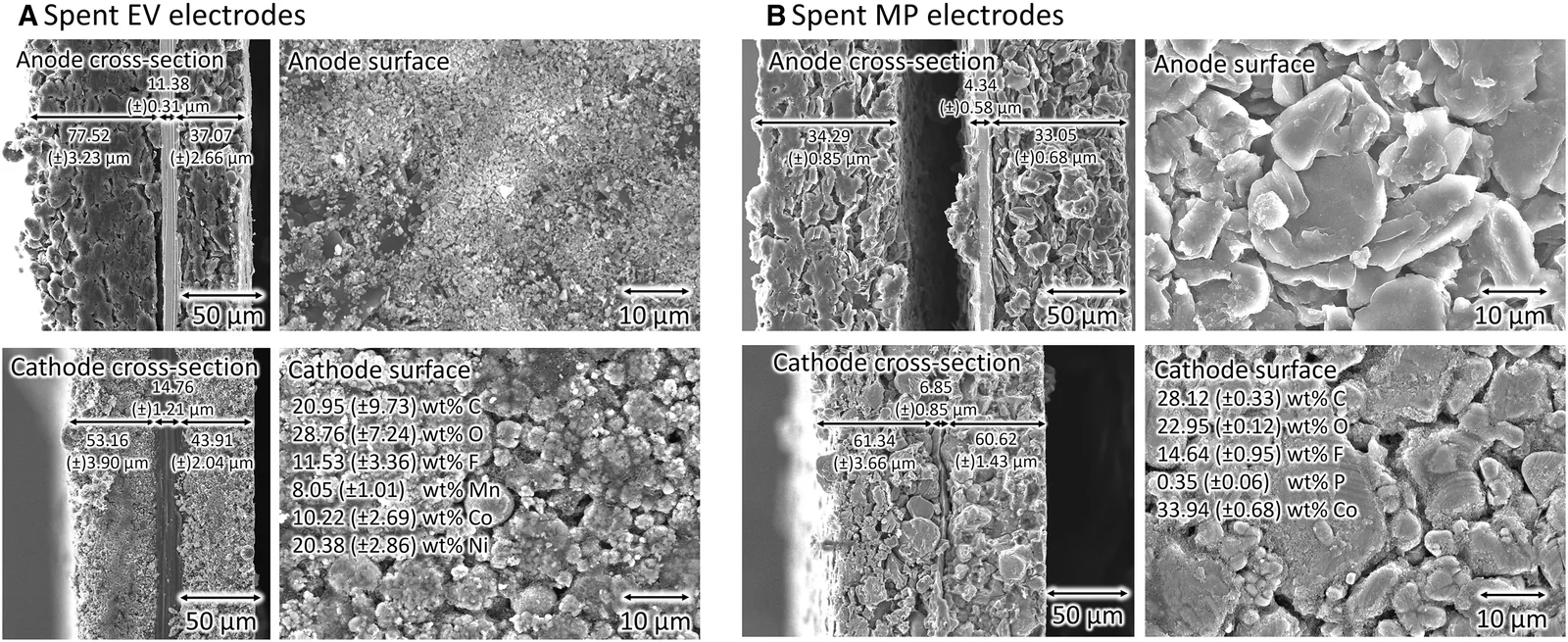
Electrode morphology SEM characterization of cathodes and anodes from (A) EV and (B) mobile phone battery. Scale bars, 50 μm (cross-section) and 10 μm (surface).

### Mechanical shredding and separation of EV module

The initial stage of the separation process involves the mechanical comminution of the EV battery modules and, separately, mobile phone cells into small pieces, targeting a nominal size of approximately <10 mm, concurrent with the physical opening of individual battery cells. Cyclone separators are commonly used in black mass purification. In this study, a slight modification to this approach was used due to the availability of equipment. After shredding, the efficient fractionation of the resultant heterogeneous mixture into its constituent components—specifically, black mass, polymer separators, and metal foils—relies critically on integrating vibratory screening and zigzag air classification. Vibratory screening (Figure 4A) facilitates the segregation of black mass particles based on significant disparities in their physical dimensions, effectively separating finer comminuted material (black mass) from larger fragments of metal foils, cell casings, and polymer separators via controlled sieve agitation. Complementarily, zigzag air classification (Figure 4B) enables the separation of materials exhibiting substantial differences in density. This is achieved by introducing a controlled upward airflow within a serpentine chamber, allowing for the differential elutriation of lighter fractions from heavier components.

**Figure 4:**
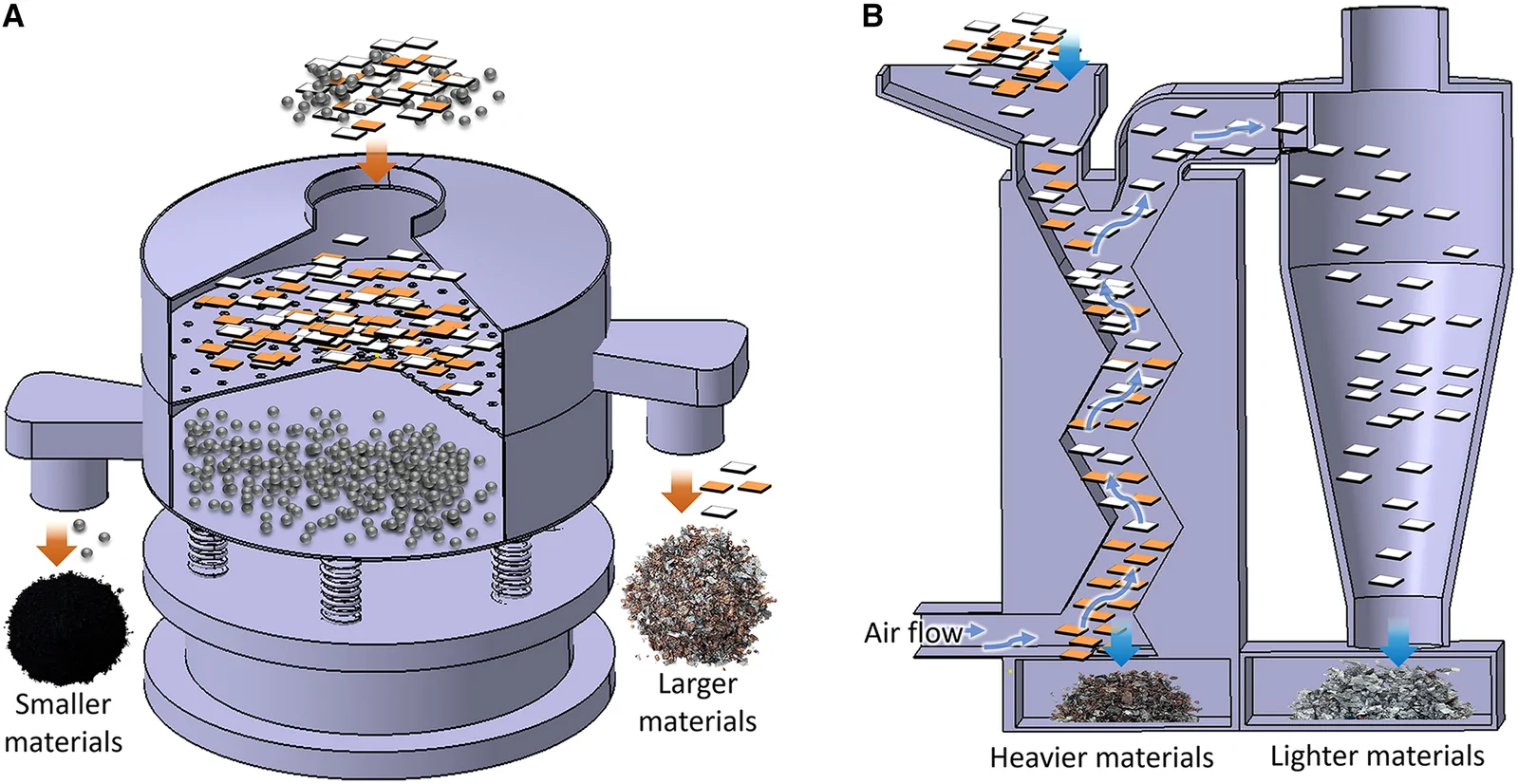
Working principles of the separation devices Illustrations of the working principle of (A) a circular vibrating screening sieve and (B) a zigzag air separator.

To facilitate black mass recovery from spent EV battery modules, a two-stage separation process is employed. The modules are initially disassembled, retaining the interconnected 16-cell assembly. This assembly is then subjected to a sequential process involving shredding, zigzag air separation, and circular screen vibration. Two distinct processing (Figure 5) routes are evaluated to optimize black mass yield. Route EV I (Figure 5A) involved a sequential process of shredding, initial circular screen vibration, zigzag air separation at motor frequencies of 23 Hz and 55 Hz, and another circular screen vibration as the final step. Route EV II (Figure 5B), on the other hand, commenced with shredding and zigzag air separation at 23 Hz, followed by two sub-routes. The first sub-route included initial circular screen vibration for lighter materials, while the second sub-route involved zigzag air separation at 55 Hz for heavier materials, followed by a final circular screen vibration. The black mass product is collected from the final stages of both routes, combining the output from the first and second passes of the circular screen vibration. The black mass separation efficiency of Route EV I and EV II is 93.28 (±4.05) wt % and 83.53 (±3.63) wt %, respectively. The quantitative analysis of the black mass separation efficiency, as detailed in the flowchart in Table 1, identified route EV I as the more efficient method for EV black mass separation. Consequently, further discussion will focus exclusively on this route. Note that the mass balance depicted in Figure 5 exhibits a potential discrepancy attributable to inherent material losses during processing. These losses encompass incomplete material retrieval from the machinery, dust entrainment within the collection system, and electrolyte volatilization during shearing. A detailed analysis of these factors, including the “lost to process” fraction, is presented in the techno-economic assessment later in discussion.

**Figure 5:**
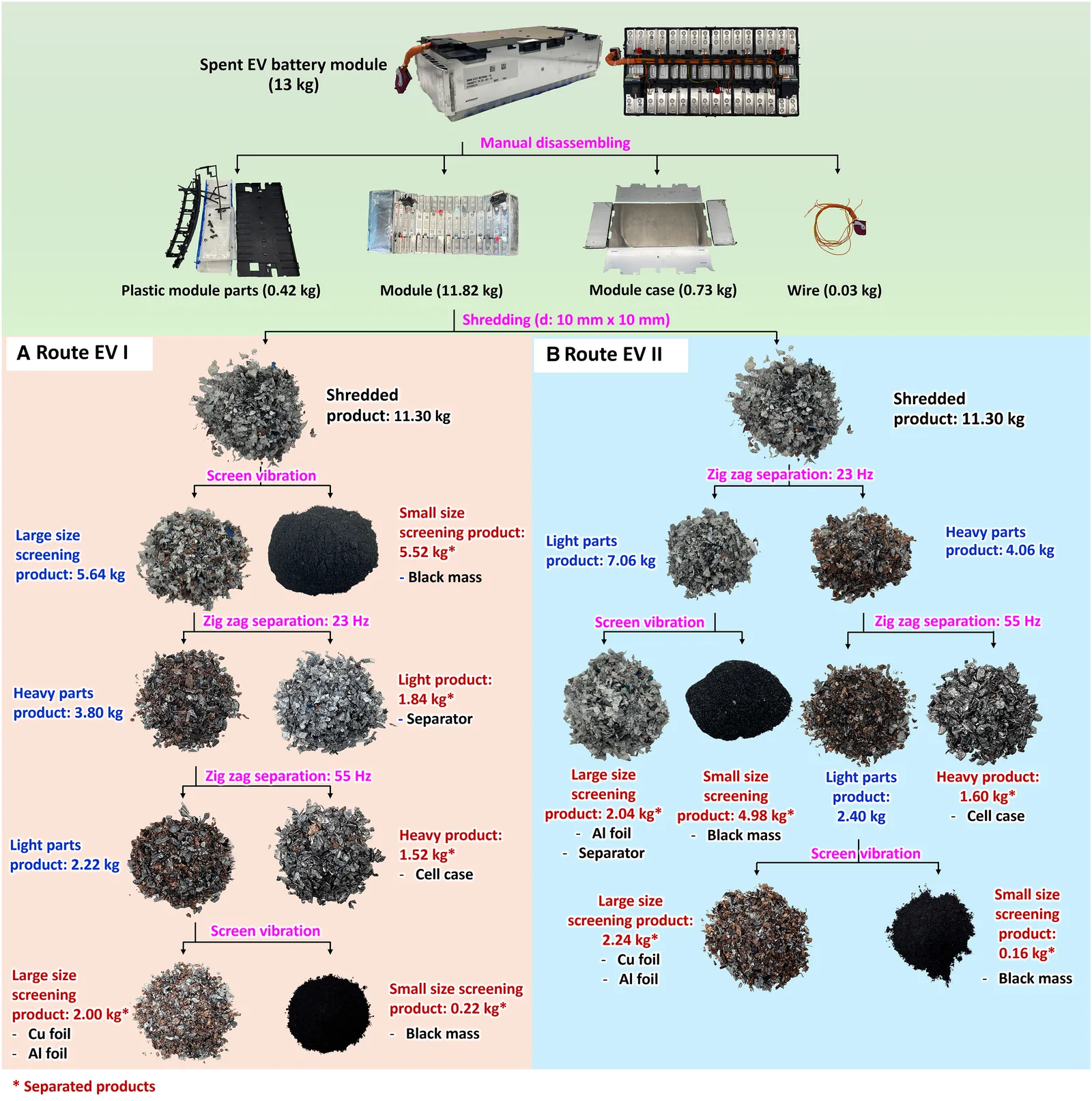
Black mass separation routes for EV battery modules Flow charts of black mass separation from the EV battery module by (A) Route EV I, involving a sequential process of shredding, initial circular screen vibration, initial zigzag air separation at 23 Hz, subsequent zigzag air separation at 55 Hz, and final circular screen vibration, and (B) Route EV II, which commences with shredding and initial zigzag air separation at 23 Hz, followed by either direct circular screen vibration or a secondary zigzag air separation at 55 Hz, followed by circular screen vibration. Mass values are mean of *n* = 3 independent batch separation runs.

**Table 1:** Baseline black mass (BM) composition, mechanical recovery performance, and financial modeling across regional feedstock market scenarios (per 1 kg raw battery input)

Battery source	Baseline BM (wt %)	Recovered BM (wt %)	Separation efficiency (%)	Product revenue ($/kg)	Loaded cost ($/kg)	Baseline net profit (zero feedstock cost) ($/kg)	Scenario A net profit (+$2–3/kg gate fee) ($/kg)	Scenario B net profit- feedstock purchase ($/kg)
EV battery	47.33 (±2.00)	44.15 (±3.78)	93.28 (±4.05)	7.20	0.31	6.89	8.89–9.89	4.39 (at $2.50/kg NCM scrap)
Mobile phone	71.16 (±4.15)	53.92 (±6.10)	75.77 (±4.20)	8.96	0.73	8.23	10.23–11.23	5.53 (at $2.70/kg LCO scrap)

Data are represented as mean (±SD); *n* = 3 independent batch separation runs. Financial metrics are point estimates derived from the corresponding mean recoveries at reference black mass prices of $14.22/kg (NMC622) and $14.05/kg (LCO); see STAR Methods.

Additionally, the shredded materials were in a dry state as the solvent evaporated during the processing phase. Typical solvents include ethylene carbonate (EC), often combined with propylene carbonate (PC) and linear carbonates such as dimethyl carbonate (DMC), diethyl carbonate (DEC), and ethyl methyl carbonate (EMC).^58^^,^^59^ A significant limitation of LiPF_6_-based electrolytes is their poor thermal stability. While extensive research often investigates this thermally driven chemical degradation at elevated temperatures, frequently as high as 85 °C, it is important to note that these degradation reactions demonstrably commence at lower temperatures (<40 °C).^58^^,^^59^ This is attributed to the electrolyte’s susceptibility to moisture absorption from the ambient environment, which triggers a cascade of undesired side reactions.^60^^,^^61^ Therefore, a nitrogen gas purging system was implemented in the shredder to prevent undesirable reactions and mitigate the release of potentially hazardous gases into the environment, with the flow directed from the base of the shredding apparatus and collected at the top.

Furthermore, the proposed flowsheet was designed to address specific limitations reported for conventional mechanical shredding and size-based multi-stage sieving approaches,^62^^,^^63^ namely separation efficiency and product purity—quantitatively benchmarked against pilot- and commercial-scale routes later in discussion (Table 2). Traditional industrial sorting routes often suffer from severe metal cross-contamination because fine copper and aluminum foil shreds match the particle size metrics of the liberated active materials^63^; moreover, residual active materials often remain adhered to the foil substrates and ancillary components. Complementarily, zigzag air classification (Figure 4B) enables the separation of materials with substantial density differences. This is achieved by introducing controlled upward airflow within a serpentine chamber, allowing for the differential elutriation of lighter fractions from heavier components. In our customized flowsheet, this aerodynamic mechanism is operated at optimized motor frequencies (23 Hz and 55 Hz) and strategically sequenced with circular screen vibration across two distinct configurations (Route EV I and Route EV II). Collecting the final black mass product by combining the outputs from the first and second passes of the final circular-screen vibration stage successfully avoids redundant, energy-intensive over-milling loops that typically increase operational costs and generate hazardous fugitive dust in standard recycling facilities.^63^^,^^79^ Consequently, this targeted sequential design balances separation efficiency and product purity while simultaneously optimizing the economic and environmental performance metrics of the primary recovery stage.^79^

**Table 2:** Benchmarking of the proposed mechanical separation flowsheet against pilot-scale and commercial black-mass production routes

Parameter	This study (route EV I/MP I)	Pilot-scale	Commercial-scale	Industry purity specification
Throughput	13 kg/2.04 kg single-batch; 100 kg/h pilot line design capacity	50–1,000 kg/h (e.g., RecycLiCo/Kemetco demonstration plant: 500–815 kg/day)^64^^,^^65^^,^^66^	3,000–500,000 + t/yr across ten named facilities (e.g., Fortum, Harjavalta: ∼3,000 t/yr; BASF, Schwarzheide: 15,000 t/yr; CATL Brunp: 500,000 + t/yr)—full list in Table S10^65^^,^^67^^,^^68^^,^^69^^,^^70^^,^^71^^,^^72^^,^^73^	–
Specific energy	0.236 kWh/kg (EV)/0.271 kWh/kg (MP) (Table S5)	not reported in comparable units in sources identified	comprehensive industrial-scale energy inventories now available^74^	–
Black mass yield (wt % of input)	44.15% (EV)/53.92% (MP)	–	reported to vary from ∼5.8% to substantially higher depending on cell design and current-collector decoating efficiency^65^	–
Product purity (Al/Cu/F)	EV: <1 wt % Al, <1 wt % Cu; MP: 0.72 wt % Al, 1.01 wt % Cu; F not quantifiable by ICP-OES	–	0.6–1.3 wt % Cu, 1.3–3.8 wt % Al reported across automotive cell types^65^	Fastmarkets NCM spec: ≤2% Al, ≤2% Cu, ≤1% Fe, ≤2% F^75^; China draft Grade 1 standard: ≤0.5% Cu, ≤1% Al, ≤0.5% Fe^76^
Processing cost	$0.11/kg (EV)/$0.53/kg (MP) direct; $0.31/$0.73 loaded	$2–6/kg typical of pilot-scale processes^77^	no independently verified mechanical-specific commercial cost figure identified—see main text for downstream-route (pyro/hydro/direct) cost and net-benefit figures instead^78^	–

### Mechanical shredding and separation of mobile phone cells

To isolate black mass from mobile phone lithium-ion battery cells, 2 kg of brine-discharged cells is directly fed into a black mass separation unit. Similar to EV cells above, two distinct processing routes are employed herein. Route MP I, shown in Figure 6A, involves sequential shredding, coarse screening, and two-stage air classification (23 Hz) with a zigzag air separator. The heavier fraction from the initial air classification is subjected to a second round of air classification at a higher frequency (55 Hz), followed by a second screening pass, while the lighter fraction undergoes a second screening pass. Route MP II, shown in Figure 6B, followed a similar sequence but with the order of air classification and screening reversed for the initial separation. Similar to the EV battery module approach, the black mass product is collected from the stages of both routes by combining the output from the first and second passes of the circular screen vibration. The black mass separation efficiency of routes MP I and II from the ca. 2 kg mobile phone battery cells are 75.77 (±4.20) wt % and 59.31 (±7.62) wt %, respectively. The MP I route, again, demonstrates significantly higher black mass separation efficiency compared to MP II. This observation is likely attributable to the triplicate screening process employed in MP I. Consequently, route MP I will be the focus of subsequent discussion due to its superior separation efficiency. Despite the observation that separating black mass from mobile phone battery current conductors poses greater challenges than EV batteries, a conclusive explanation for this difference remains unclear due to current data limitations. This disparity may arise from variations in cathode sheet composition, encompassing binder types and component ratios. Although mobile phone batteries' black mass separation efficiency is currently suboptimal, significant improvements may be achievable through pyrolysis removal of the PVDF-based binder after the shredding process. Furthermore, while brine immersion discharging effectively neutralizes small individual cells for safe shredding, the chemical residues and liquid waste treatment penalties inherent to this method introduce baseline pre-treatment variances that must be considered when directly comparing the economic and environmental performance of the MP route against the EV route.

**Figure 6:**
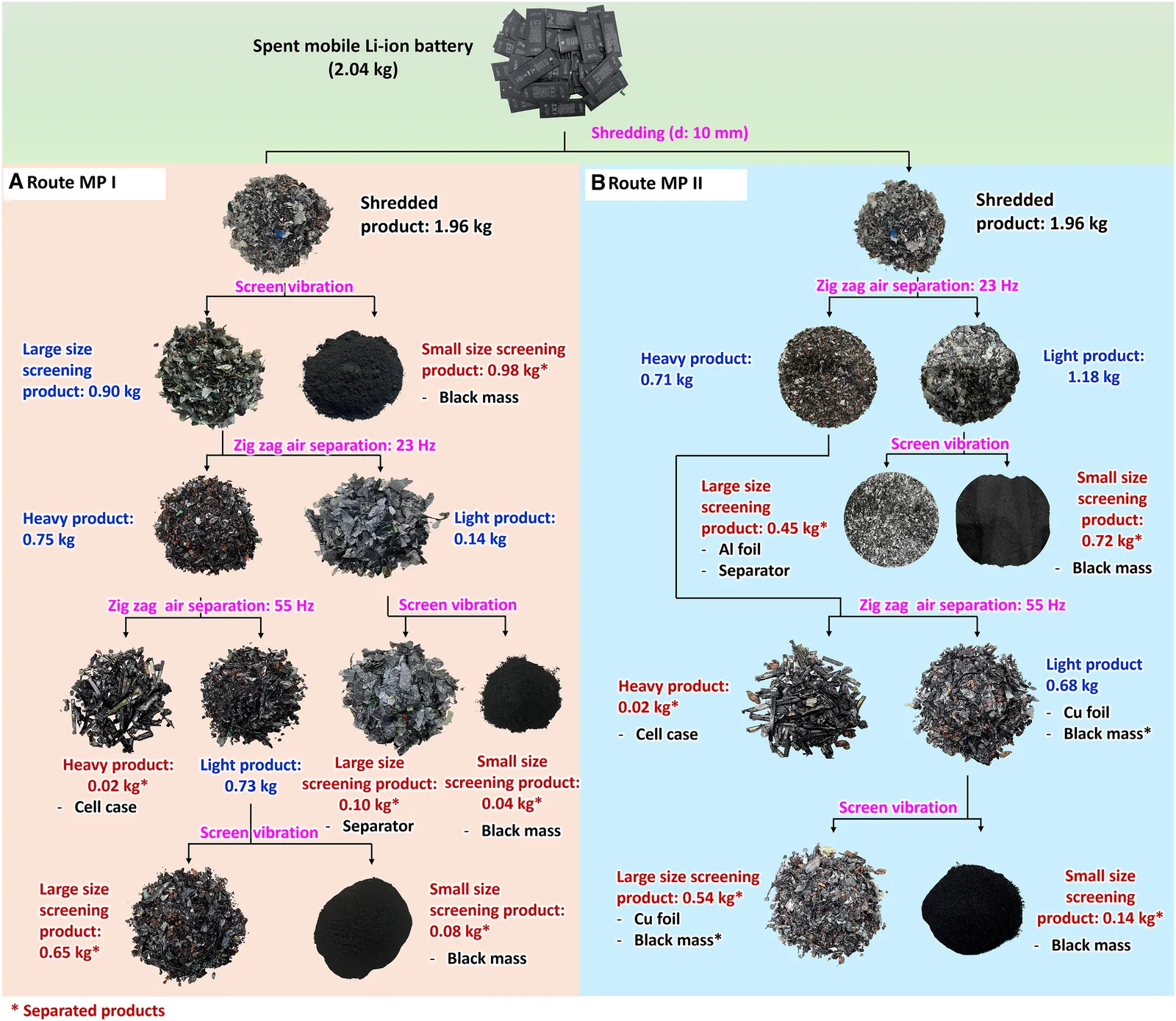
Black mass separation routes for mobile phone battery Flow charts of black mass separation from the mobile phone battery by (A) Route MP I, involving a sequential process of shredding, initial circular screen vibration, initial zigzag air separation at 23 Hz, subsequent zigzag air separation at 55 Hz, and final circular screen vibration, and (B) Route MP II, which commences with shredding and initial zigzag air separation at 23 Hz, followed by either direct circular screen vibration or a secondary zigzag air separation at 55 Hz, followed by circular screen vibration. Mass values are mean of *n* = 3 independent batch separation runs.

It is noted that enforcing a single, uniform mechanical separation workflow across both feedstocks is unviable due to the structural, dimensional, and chemical disparities between the 13 kg multi-cell EV modules and the 2 kg bulk batch of individual MP cells. As demonstrated by the cross-sectional characterization data, the distinct physical constructions and significantly varied current collector foil thicknesses—ranging from an ultra-thin 4.34–6.85 μm for MP cells to a thicker 11.38–14.76 μm for EV modules—fundamentally alter their post-shredding fragmentation geometries and aerodynamic drag profiles within the zigzag chamber. Furthermore, the spontaneous air-delamination behavior observed in the EV anode matrix contrasts with the mechanical tenacity and detachment challenges characteristic of the MP cell fractions. Consequently, to maintain strict experimental rigor and isolate parameters, the single-factor controlled-variable principle was systematically applied within each battery stream (Route I vs. Route II) rather than imposing a uniform sequence across fundamentally mismatched material baselines.

Because the proposed physical flowsheet incorporates initial size classification via circular screen vibration (35-mesh/500 μm sieve) prior to air classification, liberated cathode active oxides and anode graphite are collected simultaneously as a unified black mass powder. Crucially, the initial screening step recovers the vast majority of the total black mass—96.2 wt % (5.52 kg out of 5.74 kg total black mass product) in Route EV I and 89.1 wt % (0.98 kg out of 1.10 kg total black mass product) in Route MP I—before the material enters the zigzag classifier. Consequently, density-based air separation operates strictly on pre-screened coarse fragments (>500 μm up to 10 mm) to segregate structural cell casings, polymer separators, and metallic foils. Secondary circular screen vibration is applied selectively to foil- and separator-rich streams where fine active powders remain physically attached to substrate surfaces. Fractions bypassing secondary screening consist of clean macro-components (e.g., rigid cell casings) carrying negligible active powder. Because bulk active material is isolated by particle size screening rather than air elutriation, aerodynamic density differences between graphite (∼2.2 g cm^−3^) and metal oxides (>4.5 g cm^−3^) do not significantly cause phase-selective loss. Assuming mathematically uniform physical recovery across both active materials is therefore justified, matching the overall separation efficiencies of 93.28 wt % for Route EV I and 75.77 wt % for Route MP I.

### Compositional, structural, and quality characterization of separated black mass

Figure 7 illustrates the combined SEM micrographs, crystalline phase identification, and particle size distributions of the resultant black mass obtained after the separation processes of routes EV I (Figure 7A) and MP I (Figure 7B). SEM imaging reveals a heterogeneous particulate mixture where individual active particles are cleanly liberated but clustered. While the fundamental, original grain size of graphite and cathode particles is typically under 30 μm, sieve-size metrics indicate a dominant distribution below 125 μm, with a minor distribution in the 125–600 μm range. This larger apparent size profile is attributed to binder-mediated particle agglomeration, demonstrating that the active material retains its interparticle adhesion forces without requiring destructive, energy-intensive overgrinding. Crystalline structural integrity was confirmed via XRD analysis. As shown in the diffraction patterns, the co-existence of intact anode graphite and lithium metal oxide phases (LiNi_0.6_Mn_0.2_Co_0.2_O_2_ for EV; LiCoO_2_ for MP) matches their respective reference standards perfectly, indicating that no phase transformations or degradation occurred during mechanical sorting. Critically, ICP-OES analysis quantified the elemental composition of the EV I black mass as approximately 3.91 wt % Li, 15.02 wt % Ni, 6.01 wt % Mn, 7.41 wt % Co, and 53.7 wt % C, and the MP I black mass as approximately 4.32 wt % Li, 30.95 wt % Co, and 49.90 wt % C. Structural current-collector impurities (residual Al and Cu shreds) were restricted to trace levels in both configurations: sub-1 wt % for the EV route, and 0.72 wt % Al/1.01 wt % Cu for the MP route. Fluorine content could not be quantified by either configuration, as ICP-OES is not sensitive to F; quantification would require a dedicated technique such as an ion-selective electrode or ion chromatography analysis. From a downstream application perspective, this low impurity level meets current commercial black mass trading specifications, which typically require Al, Cu, and F contents each below approximately 2 wt %.^74^ However, for direct cathode re-synthesis and regeneration pathways, residual PVDF binder (1.0–2.5 wt % F content) and cross-mixed anode graphite interfere with cathode re-lithiation and structural restoration.^80^^,^^81^ Consequently, utilizing this black mass in direct recycling requires an intermediate thermal pyrolysis step (500°C–700 °C) to burn off organic binder residues, highlighting that while the physical separation product is immediately optimal for hydrometallurgy, binder decomposition remains necessary for direct restoration routes.^81^^,^^82^

**Figure 7:**
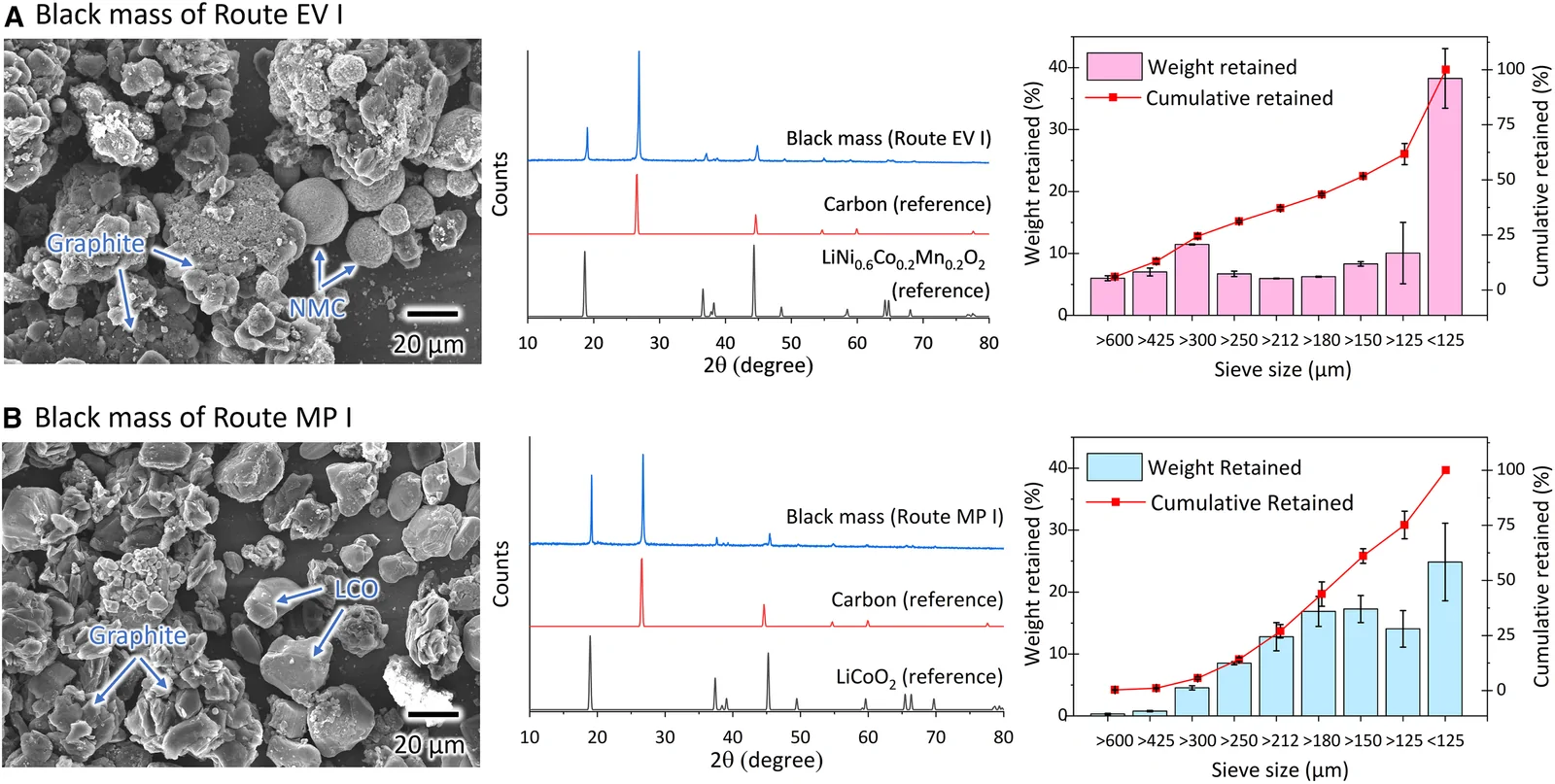
Characterization of the separated black mass Characterization of separated black mass from routes EV I (A) and MP I (B), including SEM micrographs (scale bars, 20 μm), XRD patterns, and particle size distributions. Particle size distribution data are represented as mean ± SD; *n* = 3 independent batch separation runs. See also Tables S3 and S4.

### Techno-economic assessment

Techno-economic analysis of material flow for the separation of black mass from spent EV and MP batteries, based on a 1 kg raw material input, is presented in Figure 8. This includes key consumables such as electricity, nitrogen gas, and chemical reagents. Granular details regarding average material balances and projected pricing are further elaborated in Tables S3 and S4. The material breakdown for the EV battery separation pathway (Figure 8A) yielded approximately 46.74 wt % black mass, alongside significant fractions of copper and aluminum foils (16.29 wt %), polymer separator (14.98 wt %), cell case (12.38 wt %), module case (5.94 wt %), and plastic module components (3.42 wt %). These values are typical for these types of cells.^83^ In contrast, the MP battery separation route (Figure 8B) resulted in a higher proportion of black mass (58.82 wt %) and copper/aluminum foils (34.76 wt %), with comparatively lower percentages of polymer separator (5.35 wt %) and cell case (1.07 wt %). Notably, the MP route lacked module case and plastic module parts as the input material consisted of individual battery cells, unlike the EV route, which processed entire dismantled modules. Economic projections (Table 1) indicate substantial processing profitability for both routes, with estimated profit margins ranging from 91.9% to 95.7% of the total revenue. Intriguingly, the MP route demonstrated a higher profit margin despite a lower black mass separation efficiency (ca. 75.77 wt %) compared to the EV route (ca. 93 wt %), as shown in Table 1. This disparity is attributed to the higher relative weight of black mass and electrode-associated metal foils per kilogram of raw material in MP batteries. Conversely, the processing cost for the MP route is elevated compared to the EV route, primarily due to the utilization of a NaCl salt solution for discharge, necessitating associated disposal costs. This highlights a trade-off between material composition, separation efficiency, and process economics in the black mass separation of different battery types and discharge techniques. Although aqueous solution-based immersion (10 wt % NaCl) represents a straightforward laboratory setup with low capital requirements, its long-term operational costs are substantial ($0.53/kg). This inflation is primarily driven by the depletion of chemical consumables (refined salt and fresh water) and the generation of highly corrosive, metal-contaminated liquid effluent, which incurs a substantial hazardous waste disposal fee of $0.30/kg. By contrast, the custom-engineered resistance-based discharging framework (Figure S1) operates as a closed, non-consumable system that dissipates residual capacity as thermal energy, keeping the baseline operational cost to a trace amount of electrical consumption ($0.11/kg). It is critical to recognize that applying distinct discharging methodologies across the two battery feedstocks introduces a baseline methodological variance that impacts direct TEA and LCA comparability. Brine immersion was implemented for loose mobile phone cells due to the practical difficulty of individually wiring small commercial cells to electrical resistors. However, the resulting chemical consumable requirements and hazardous liquid waste treatment fees artificially inflate the processing cost ($0.53/kg vs. $0.11/kg) and environmental impacts (notably ecotoxicity) of the mobile phone pathway relative to the EV module route. Therefore, while each discharging method reflects practical constraints for its specific feedstock scale, direct cross-route comparability must account for this initial pre-treatment discrepancy. Additionally, average prices of $14.22/kg for spent NMC622 and $14.05/kg for spent LCO were utilized in calculations in this study, derived as described in the STAR Methods.^84^^,^^85^^,^^86^^,^^87^^,^^88^ Given the substantial, multiply corroborated price recovery in battery raw materials since early 2025—cobalt prices rose approximately 160% from their February 2025 low,^89^ and NCM black mass payables reached record highs in January 2026, explicitly attributed to this rally^90^—a Monte Carlo sensitivity analysis (methodology, STAR Methods) across price and four other key variables is therefore presented later in discussion, rather than relying on a single point estimate. It is acknowledged that the black mass recovered from the EV batteries, characterized by an approximate Ni:Mn:Co atomic ratio of 0.52:0.22:0.26, does not strictly match the NMC622 stoichiometry. However, the NMC622 price was used as a comparable and competitive market benchmark for financial modeling. The reported processing costs in this study, based on price averages in Thailand, include energy, chemical reagents, and waste disposal. It is clear that the profitability of a recycling process will have a marked geographic influence. The direct variable processing cost—electricity, N_2_ gas, and (for the MP route) NaCl, water, and waste disposal—was $0.11/kg for EV modules and $0.53/kg for MP cells (Tables S3; S4). As with previous techno-economic assessments of battery recycling, several cost categories remain intangible without a working commercial-scale plant: battery collection and transport, manual pack-to-module or cell disassembly labor and working capital.^77^ A gross approximation of the order of magnitude of plant-level costs can nonetheless be made using an established model for a lead-acid battery recycling plant, which yields an estimated operational cost in the range of $0.72–$1.08/kg.^91^ To formalize a comparable baseline for the proposed mechanical-aerodynamic flowsheet specifically, a customized fixed-overhead addition was calculated: a standard 10-year straight-line depreciation matrix for the pilot-scale sorting infrastructure,^92^ localized sorting-line operator labor rates in Thailand, and a standard 15% plant management overhead multiplier^93^ together give a uniform overhead of $0.20/kg (Methods S1 and Table S6), applied identically to both routes since it reflects a plant-level throughput allocation rather than a route-specific cost. Adding this overhead to the direct processing cost gives a total loaded processing cost of $0.31/kg for EV modules and $0.73/kg for MP cells—well below the $0.72–$1.08/kg lead-acid benchmark for the EV route, and within the lower end of that range for the MP route. Subtracting the loaded processing cost from product revenue gives a baseline net profit—assuming zero feedstock acquisition cost—of $6.89/kg for EV modules and $8.23/kg for MP cells. This zero-cost feedstock baseline is not merely a theoretical reference point: the batteries used in this study were themselves obtained as donated post-consumer waste, so it reflects the actual sourcing conditions of this work, while the two scenarios below model feedstock acquisition cost under realistic commercial conditions.

**Figure 8:**
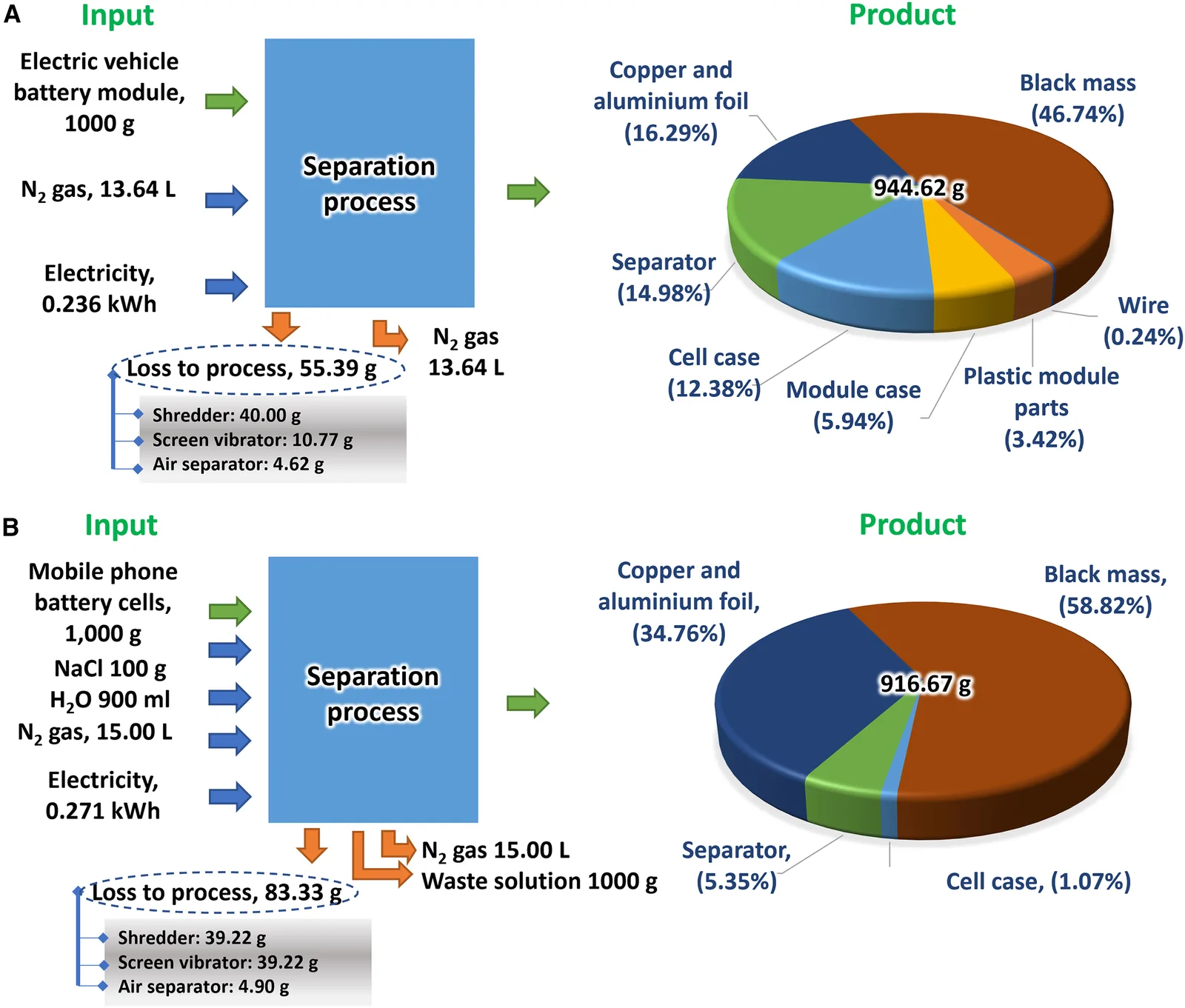
Material balance of black mass separation Material balance for electric vehicle (a, route EV I) and mobile phone (b, route MP I) battery black mass separation, per 1 kg of initial raw battery. Values are mean wt % of *n* = 3 independent batch separation runs; see also Tables S3, S4, S5.

To establish a financial framework that accounts for regional market variations, this section modeled two distinct feedstock acquisition scenarios. Under Scenario A (Gate-Fee Model), reflecting European and UK extended producer responsibility (EPR) arrangements in which recyclers are paid $2.00–$3.00/kg to accept spent LIB packs,^94^^,^^95^ gross revenue rises to $9.20–$10.20/kg for EV modules and $10.96–$11.96/kg for MP cells, driving net profit margins to $8.89–$9.89/kg and $10.23–$11.23/kg, respectively. Under Scenario B (Feedstock Purchase Model), reflecting Asian markets where spent battery scrap is traded as a positive-value commodity rather than a fee-bearing waste, recyclers must instead purchase feedstock on the open market. A Fastmarkets assessment of battery scrap prices into Southeast Asian collection hubs placed NCM-type EV scrap at approximately $2,500/tonne ($2.50/kg) and LCO-type MP scrap at approximately $2,700/tonne ($2.70/kg), depending on state of health, cell casing mass, and contained metal payables.^96^ Applying these feedstock costs to the baseline net profit above gives net operating profit margins of $4.39/kg for EV modules and $5.53/kg for MP cells—confirming the flowsheet remains viable even under realistic commercial purchasing conditions, with feedstock costs well below the $6.89/kg/$8.23/kg break-even feedstock price. This assessment reflects 2023 market conditions; more recent reporting indicates battery-scrap prices into Southeast Asia have since risen amid tightening global supply and strengthening Asian demand,^89^^,^^90^ so the margins above should be read as a conservative estimate relative to current conditions. Figure S7 presents a tornado sensitivity analysis for both routes, and Figure S8 presents the corresponding Monte Carlo simulation. Feedstock acquisition cost is the dominant driver of profitability for both routes, followed by black mass/contained-metal price; separation efficiency and plant utilization have a comparatively modest effect, and electricity price has a negligible effect at this study’s scale. Under combined uncertainty across all five variables, median net profit is $4.97/kg (EV) and $6.28/kg (MP), with a 5th–95th percentile range of $1.79–$8.61/kg (EV) and $2.70–$10.18/kg (MP). The probability of the process being unprofitable under this combined uncertainty is 0.2% (EV) and <0.1% (MP). The breakeven feedstock price is itself a distribution rather than a fixed value: mean $6.59/kg (EV, 90% CI $4.53–8.54/kg) and $7.89/kg (MP, 90% CI $5.31–10.36/kg)—the current feedstock purchase assumption ($2.50–2.70/kg) does not approach unprofitability in any simulated scenario at prevailing black mass prices.

The economic and technical feasibility of the proposed mechanical separation approach must be assessed in the context of its intended role as a pre-treatment for mainstream recycling routes, specifically pyrometallurgy, hydrometallurgy, or direct regeneration, as mechanical black-mass separation cannot efficiently recover individual elements or directly resynthesize cathode material. Therefore, the economic viability of this method may be compared relative to three established lithium-ion battery (LIB) recycling pathways. Current industrial methods include pyrometallurgy, which, despite its ability to handle bulk material and recover Ni and Co at high temperatures (up to 1700°C), is severely hindered by high energy demands and the costly loss of Li and Mn into slag, achieving a comparatively modest net economic benefit of $2–$8/kg.^97^ Hydrometallurgy, the prevailing industrial standard, operates at lower temperatures (40°C–95°C) to achieve higher overall elemental recovery but incurs substantial costs and environmental liabilities from high reagent consumption and wastewater generation, nonetheless achieving the highest net economic benefit of the three pathways at $10–$18/kg.^97^^,^^98^ In contrast, direct recycling, focusing on cathode structure preservation for high recovery, achieves a net economic benefit of $5–$10/kg, although its commercial maturity remains a challenge when processing mixed chemistries.^97^ The mechanical black mass separation approach, utilizing advanced crushing, sieving and density separation,^35^ may offer two key strategic advantages: First, its cost efficiency and low capital investment ($0.31/kg for EV modules, $0.73/kg for MP cells, loaded—see Table 2) compare favorably with reported pilot-scale processing costs of $2–$6/kg.^77^ Furthermore, it provides a logistical advantage by efficiently handling diverse, mixed battery waste streams without extensive manual pre-sorting.^99^^,^^100^ Second, the methodology ensures optimized black mass purity; the robust separation achieved here delivers a high-grade cathodic concentrate, which is paramount for maximizing the efficiency and cost-effectiveness of subsequent hydrometallurgical leaching or direct regeneration steps.^101^

Table 2 quantifies this comparison explicitly, benchmarking the present flowsheet against pilot- and commercial-scale black-mass production routes (full facility listing in Table S10). The present work operates at laboratory/pilot demonstration scale (13 kg EV, 2.04 kg MP single-batch runs; 100 kg/h pilot design capacity). This sits below even the smallest commercial black-mass operation identified in a survey of ten named facilities (Fortum, Harjavalta: ∼3,000 t/yr) and within the pilot/demonstration throughput band (50–1,000 kg/h) rather than the commercial band (3,000–500,000 + t/yr) spanned by those facilities. The specific energy achieved here (0.236–0.271 kWh/kg) and black mass purity (sub-1 wt % Al/Cu for the EV route; 0.72/1.01 wt % Al/Cu for the MP route) compare favorably with reported values for mechanically produced black mass at any scale and meet current commercial buyer specifications (e.g., Fastmarkets' ≤2% Al/Cu/F thresholds), but this should be read as a proof-of-concept baseline rather than a validated substitute for industrial performance—zigzag classification efficiency in particular is known to degrade with increasing solids loading, a scale-dependent effect this study’s batch scale cannot characterize. What is new here is not throughput but methodology: a fully paired techno-economic and life cycle baseline for the same physical flowsheet applied to two structurally distinct feedstocks (EV modules vs. mobile phone cells) under identical costing and impact-assessment boundaries—a direct EV-vs-consumer-electronics comparison that, to our knowledge, does not yet exist at either pilot or commercial scale in the published literature.

Additionally, acknowledging the increasing market penetration of Lithium Iron Phosphate (LFP) batteries—driven by cost, safety, and the absence of scarce Ni and Co—the current study prioritized NMC due to its immediate high-value criticality.^97^ The lack of high-value metals in LFP necessitates a distinct, cost-focused recycling strategy, as conventional pyrometallurgy and hydrometallurgy are often unprofitable for LFP, making its economics highly sensitive to lithium market volatility.^97^ Despite this fundamental economic divergence, the proposed low-cost mechanical pre-treatment is strategically suitable for LFP processing, aligning with the primary goal of cost minimization. However, the subsequent refinement of LFP black mass differs fundamentally from NMC: LFP processing shifts away from complex Ni/Co/Mn solvent extraction toward efficient lithium extraction or direct cathode regeneration.^14^ While mechanical black mass separation can be an effective precursor for lithium extraction, its suitability for direct regeneration—the most environmentally preferred LFP pathway—is contingent upon achieving extremely high-purity spent cathode material completely free of anode contamination, a requirement that may challenge current mechanical separation capabilities.

### Life cycle analysis

A life cycle assessment was also carried out using the same inventory data as the techno-economic assessment of the EV and MP battery treatment pathways to highlight the environmental impact of the different processes and ensure these impacts are also considered when deciding on an optimal black mass production procedure. The same scope and functional unit as the techno-economic assessment were used: the treatment and separation of 1 kg of incoming battery waste into its constituent components to produce a mixed black mass. Additionally, the environmental impact per module will also be given for reference. The primary environmental impact metric quoted in this study is the global warming potential (GWP), using the IPCC 2021 methodology.^102^ But additional metrics, which were considered, include water toxicity, land usage, and water scarcity, using methodologies outlined by the European Union and weighted according to their proximity to EU 2020 policy targets.^103^^,^^104^ These EU-centric methodologies were used due to their applicability to real-world policy targets and the fact that similar Asia-based frameworks are not available on the software used to carry out this life cycle assessment. The definitions of each metric are given in Methods S2. Each process in this study (Route EV I & II, Route MP I & II) were modeled and assessed using Umberto 11 software with an Ecoinvent 3.11 database to supply secondary data relating to solvent production, electricity generation, and potential waste treatments. Data regarding electricity usage were modeled with regard to the electricity production mix in Thailand from 2022.^78^^,^^105^ The datasets used to model the electricity usage, as well as the other datasets used in this study, are given in Table S7.

Table 3 outlines the data acquired from this study with regard to each of the environmental impact metrics. Route EV I and MP I were compared, along with additional scenarios considering the treatment of waste and relatively low-value products using standard treatment processes from the Ecoinvent 3.11 database. The life cycle inventory for Route EV I and Route MP I, detailed in Methods S2 and Table S8, includes all phase-by-phase inputs and outputs related to electricity consumption, chemical reagents, and solvents, providing the basis for the calculations in Table 3. Initially, it can be observed that the environmental impacts of the processes themselves, per kg of battery waste, are comparable when treating either an EV or MP battery module. The EV I route, with shredding contribution added, gives a GWP of 0.210 kg CO_2_ equiv. and the MP I route giving 0.241 kg CO_2_ equiv. This result is largely driven by the similar electricity usage of 0.236 kWh and 0.271 kWh, which is responsible for the majority of the impact metrics without accounting for waste treatments. When looking at the results per module, this similarity is lost due to the size disparity between the different modules, but it does give a more complete view of the environmental impacts of the processes described in this work.

**Table 3:** Environmental impact results relating to Route EV I and Route MP I per 1 kg of initial raw battery calculated using Umberto 11 and the Ecoinvent 3.11 database for upstream and waste processes. Scenarios, including waste processing, are denoted with “W”

Environmental impact metric	Unit/1 kg input	Route EV I	Route MP I	Route EV I-W	Route MP I-W
Global warming potential (GWP)	kg CO_2_ equiv.	0.210	0.241	0.240	2.377
Freshwater ecotoxicity	CTUe	0.406	0.464	0.638	16.883
Land use	Points	0.169	0.193	0.372	1.316
Water scarcity	m^3^ world equiv.	0.027	0.031	0.033	0.140

As the majority of the impact of the process itself derives from electricity usage, electricity production in Thailand becomes a key factor in the environmental footprint of the process. From Table S7, it can be seen that over 75% of the electricity production is from relatively high-carbon sources, namely gas and coal. This will significantly increase the GWP/kWh of electricity produced when the process is done in Thailand, compared to countries with a greater amount of renewable energy infrastructure, such as solar and wind power. For example, while Thailand only had around 4% of its electricity from wind and solar in 2022, the UK produced over 30% of its electricity from these sources.^93^ As shown in Table S9, relocating the EV battery pretreatment process to a low-carbon grid reduces the GWP by 62.8%, dropping from 0.210 kg CO_2_ equiv./kg input (0.476 kg CO_2_ equiv./kg black mass) to 0.078 kg CO_2_ equiv./kg input (0.177 kg CO_2_ equiv./kg black mass). Parallel reductions are observed in freshwater ecotoxicity, land use, and water scarcity metrics. This scenario analysis underscores that physical mechanical separation inherently possesses a low direct carbon footprint, and its life cycle environmental performance automatically scales down as global and regional energy grids transition toward carbon neutrality. This scenario is illustrated for the EV route; the same grid-dependency principle applies proportionally to the MP route, whose GWP is similarly dominated by electricity consumption during separation.

Similarly, water consumption relating to conventional gas and coal power emissions can be significant due to the cooling processes needed in conventional plants, which are not as prevalent in alternative forms of energy generation.^106^ In the case of EV I, to treat one module (13 kg), the equivalent of 0.354 m^3^ (354 L) of water is consumed in the generation of the necessary electricity. Both of these metrics show the significant impact electricity generation can have on the overall environmental footprint of a process and also emphasize the importance of location due to differences in energy makeup. However, it should be noted that the aquatic ecotoxicity metrics, measured on a 1–100 CTUe scale, for both EV I and MP I are relatively low, never surpassing 10 CTUe (except for the MP I-W scenario). It should be noted that CTUe, along with the other metric units, are defined in the SI. This will likely be related to the water consumption in these instances, being for cooling processes in energy generation, with minimal contamination from processing that would be hazardous to marine life.

However, when the impact of waste treatments is considered, the increased environmental impact of the MP routes compared to the EV ones is particularly significant. This is primarily due to the treatment of the waste NaCl solution from the discharge step, with the dataset used from Ecoinvent contributing significantly to all impact factors, notably the GWP and water toxicity. Like with the rest of the waste treatment datasets, the waste solution is assumed to be incinerated after use for ease of processing at scale. Therefore, environmental metrics would also be reduced significantly if waste solutions were reused or reconditioned before further reuse. In this instance, the impact of the discharge step in the MP routes could be reduced by approximately 45% for both impact factors if the discharge solutions are reused for another module, increasing to a 60% reduction if reused for an additional time. The waste treatment scenarios also assume that some products are collected and incinerated for energy recovery, including the cell casings, wiring, and copper and aluminum foils. The impacts of these other waste treatments are relatively minimal as they deal with much smaller quantities than the waste solution. However, it should be noted that some of these “waste” products may be recovered and reused in some recycling schemes, especially the current collector foils, but this is not assumed in this study. This represents a deliberately conservative choice for the environmental assessment: these same streams are modeled as sold products in the techno-economic analysis (STAR Methods), where they represent 12.8% of EV revenue and 15.3% of MP revenue. The two analyses are therefore not run on identical end-of-life assumptions for these specific streams, reflecting their differing materiality—financially significant, but (as shown above) environmentally minor relative to the waste-solution treatment that dominates this study’s impact profile. The “lost to process” material fraction identified in Figure 8 (5.5–8.3 wt % of input, arising from incomplete material retrieval, dust entrainment, and electrolyte volatilization during shredding) is not assigned any environmental burden in the current life cycle inventory. This fraction plausibly contains hydrogen fluoride, from hydrolysis of residual LiPF_6_ electrolyte salt—the reason for the N_2_ purge during shredding described in STAR Methods—alongside volatile organic compounds from carbonate-based electrolyte solvents and fine particulate matter. Fugitive emissions of this type have been characterized at operating spent-LIB recycling facilities elsewhere, with reported VOC concentrations of 54–323 mg/m^3^ in exhaust air.^107^ However, no mass-based emission factor specific to mechanical pretreatment of untreated (non-pyrolysed) spent LIBs, suitable for direct incorporation into an Ecoinvent-based inventory, was identified in the published literature or measured in this study. The reported environmental impacts should therefore be interpreted as excluding this fugitive emissions pathway, representing a conservative lower bound rather than a complete inventory.

The data collected in this study emphasize how circularity in recycling processes and the location in which waste treatments are undertaken can have a significant impact on the overall environmental impact. In both the EV and MP scenarios, the relatively high-carbon energy mix of Thailand and the infrastructure that is needed to generate this power, such as cooling towers, are the primary contributors to the LCA metrics, notably GWP and water scarcity metrics. Furthermore, in the waste treatment scenarios, it becomes clear that recovery and reuse of all battery components, rather than treating them as waste, could significantly reduce environmental impacts in all considered instances. Overall, improving separation procedures to avoid contamination that may prohibit reuse or effective recovery of every battery component should be considered, and the present work gives a suitable baseline for comparing such novel separation and battery treatment strategies in the future.

### Strategies for improving battery disassembly and black mass separation efficiency

The initial step of spent battery disassembly should be fundamentally redefined by robotic automation, which establishes the economic and safety foundation for the recycling value chain. Manual disassembly of high-voltage EV battery packs presents severe hazards to human workers, including the risk of high-voltage injury from residual charge and exposure to toxic chemicals, significantly raising the potential for thermal runaway if the cell structure is compromised.^108^ Therefore, automation is paramount for worker safety, enabling robotic systems to manage hazardous materials and complex structures.^109^ Furthermore, automation addresses the high design variability and labor-intensive nature of EOL-EV packs, where human labor contributes substantially to costs. Efficiency gains are significant, with automated processes dramatically reducing EV pack teardown time and overall disassembly costs.^91^^,^^110^ The benefits also extend to small electronics, where automated systems achieve far greater throughput. Ultimately, automation is critical for maximizing material recovery, as it enables the precise separation and sorting of components, which directly improves the purity and value of the black mass feedstock for subsequent physical and chemical recycling processes.^110^^,^^111^ Accordingly, the adoption of automated disassembly systems is strongly recommended for large-scale, industrial battery recycling initiatives.

Despite the achieved black mass separation efficiencies of 93% for the EV route and 76% for the MP route, further enhancement is possible. Note that these separation efficiencies were benchmarked against the baseline obtained by separating cathode and anode materials via manual dismantling and separation, as discussed at the start of this section. A critical factor influencing black mass separation efficacy remains the polymeric binder, which irreversibly binds the active electrode materials. Consequently, future research should prioritize strategies to disrupt, remove, or better redesign the binder chemistry so as to allow simple water delamination to facilitate improved black mass liberation.^40^^,^^112^^,^^113^^,^^114^ For instance, implementing a thermal pre-treatment step involving pyrolysis at approximately 150°C–700°C could effectively degrade the binder’s structural integrity.^115^^,^^116^ This is supported by the findings presented in Figures 1A and 1B, which illustrate the attainment of clean electrode metal foils after pyrolysis treatment at 500°C for 1 h.

From a process engineering standpoint, the sieve screening stage presents opportunities for optimization. While crucial for separating black mass, the methodology in this study necessitates two to three sequential passes. For industrial scalability and efficiency, a single-stage separation is desirable. Enhancements to the sieve screen vibration process could potentially improve black mass separation efficiency. This may involve a systematic optimization of machine parameters, including adjusting the vibration frequency, increasing the sieve diameter to prolong the residence time of shredded materials on the screen, and reducing the material feeding rate to minimize the agglomeration and stacking of battery particles, thereby promoting more effective separation.

While this study focuses on the separation of black mass from battery modules and cells, as they are the most valuable components of the battery cells, it is crucial to acknowledge the significance of recovering and recycling other components, such as separators, aluminum, and copper. These materials can be reintroduced into the industrial value chain through various separation techniques. For instance, eddy current separation can effectively isolate aluminum, copper, and other plastics.^30^ Utilizing differences in conductivity and variable magnetic fields can change the trajectory of nonferrous metals, thereby facilitating their separation from other materials.^117^

In this study, the technical feasibility and efficiency of black mass separation from spent lithium-ion batteries were investigated, focusing on industrial scalability using both electric vehicle modules and mobile phone cells as representative battery sources. Two distinct separation routes were evaluated for each battery type, employing a custom-designed separation unit comprising a shredder, a zigzag air separator, and a circular screen vibrator machine.

For EV batteries, route EV I, involving sequential shredding, initial circular screen vibration, zigzag air separation, and final circular screen vibration, demonstrated superior black mass separation efficiency (93.28 ± 4.05 wt %) compared to route EV II (83.53 ± 3.63 wt %). Similarly, for mobile phone batteries, route MP I, utilizing sequential shredding, coarse screening, and two-stage air classification, yielded a higher black mass separation efficiency (75.77 ± 4.20 wt %) than route MP II (59.31 ± 7.62 wt %). The resultant black mass from EV batteries (route EV I) comprised approximately 3.91 wt % Li, 15.02 wt % Ni, 6.01 wt % Mn, 7.41 wt % Co, and 53.7 wt % C, with trace amounts of Al and Cu. The black mass from mobile phone batteries (route MP I) similarly comprised approximately 4.32 wt % Li, 30.95 wt % Co, and 49.90 wt % C, with 0.72 wt % Al and 1.01 wt % Cu, consistent with an LCO-graphite mixture. Economic analysis indicated substantial baseline profitability for both routes: assuming zero feedstock acquisition cost, net profit margins were 95.7% of revenue for EV modules and 91.9% for MP cells. In absolute terms, the MP route generated a higher profit per kilogram of raw material ($8.23/kg vs. $6.89/kg) despite its lower separation efficiency, reflecting the greater relative mass of black mass and electrode metal foils recovered per kilogram of MP feedstock—though as a proportion of revenue, EV’s margin is actually the higher of the two. Under realistic open-market feedstock purchase prices, both routes remained profitable, at $4.39/kg (EV) and $5.53/kg (MP).

While the achieved black mass separation efficiencies are promising, further enhancement is possible. The polymeric binder, which cohesively binds active electrode materials, presents a key challenge. Future research should prioritize strategies to disrupt or remove this binder, such as implementing a thermal pre-treatment step involving pyrolysis (e.g., at 150°C–700°C), as demonstrated by the clean electrode metal foils obtained after pyrolysis at 500°C for 1 h in this study. Additionally, optimizing the sieve screening stage, potentially through adjustments to vibration frequency, sieve diameter, and reduced material feeding rate, could improve black mass separation efficiency and streamline the process for industrial applications. Beyond black mass recovery, future work should address the recovery and recycling of other valuable battery components, including separators, aluminum, and copper, to maximize resource utilization and minimize environmental impact.

### Limitations of the study

This study characterizes single-batch runs (13 kg EV, 2.04 kg mobile phone) on a 100 kg/h pilot design. Zigzag classification efficiency is known to degrade with increasing solids loading—a scale-dependent effect that cannot be characterized at this batch scale—so reported efficiencies should be read as a proof-of-concept baseline rather than validated industrial performance. The two routes also employ different discharge methods (electrical resistor discharge for EV modules; brine immersion for mobile phone cells), which inflates the processing cost and ecotoxicity attributed to the mobile phone route and limits direct cross-route comparability. The techno-economic boundary covers the mechanical separation step only; battery collection and transport, pack-to-module disassembly labor, and working capital are excluded. The life cycle inventory assigns no burden to the 5.5–8.3 wt % “lost to process” fraction, as no mass-based fugitive-emission factor for non-pyrolyzed mechanical pretreatment exists in the literature, making the reported impacts a conservative lower bound. Fluorine content could not be quantified by ICP-OES, so compliance with fluorine-specific impurity thresholds could not be assessed. Finally, each route was assessed for a single cathode chemistry—NMC622 for EV modules and LCO for mobile phone cells—so the findings may not extend directly to other chemistries such as LFP. The comparatively thin and volatile LCO black mass market, evidenced by its markedly wider single-day trading spread, adds further uncertainty to the mobile phone route’s revenue estimates.

## Resource availability

### Lead contact

Further information and requests for resources should be directed to and will be fulfilled by the lead contact, Sakhob Khumkoa (sakhob@sut.ac.th).

### Materials availability

This study did not generate new unique materials. The spent battery feedstocks were obtained as donated post-consumer waste as described in the method details.

### Data and code availability

• All data supporting the findings of this study are included in the article and its supplemental information; source data for the techno-economic and life cycle calculations are provided in Tables S3–S9.
• The Python scripts used for the tornado and Monte Carlo sensitivity analyses are available from the lead contact upon request.
• Any additional information required to reanalyze the data reported in this paper is available from the lead contact upon request.

## Acknowledgments

The authors would like to thank the Department of Primary Industries and Mines, Thailand, the Innovative Processing and Recycling of Metals Research Centre at Suranaree University of Technology, Thailand, and the 10.13039/100017146Faraday Institution (Faraday Institution grant code FIRG057, project website https://relib.org.uk).

## Author contributions

Conceptualization, T.Y. and S.K.; methodology, T.Y., C.K., and C.L.; investigation, T.Y., C.K., and C.L.; formal analysis, T.Y. and S.S.; resources, T.P. and S.K.; writing – original draft, T.Y.; writing – review and editing, S.S., J.M.Y., T.P., A.P.A., and S.K.; visualization, T.Y. and C.K.; supervision, A.P.A. and S.K.; project administration, S.K.; funding acquisition, S.K.

## Declaration of interests

The authors declare no competing interests.

## STAR★Methods

### Key resources table

**Table:** 

REAGENT or RESOURCE	SOURCE	IDENTIFIER
**Chemicals, peptides, and recombinant proteins**		

Sodium chloride (refined salt, 97.5%)	TRSR (Thailand)	N/A
Nitrogen gas (N_2_, industrial grade, inert shredding atmosphere)	Air Liquide (Thailand)	N/A

**Deposited data**		

Techno-economic and LCA source data	This paper	Tables S3–S9; Methods S1 and S2

**Software and algorithms**		

Umberto 11	ifu Hamburg GmbH	https://www.ifu.com/umberto
Ecoinvent database (v3.11)	Ecoinvent Association	https://ecoinvent.org
Python 3 with NumPy	Python Software Foundation	https://www.python.org

**Other**		

Spent EV battery modules (NMC622, 13 kg)	Donated post-consumer waste	N/A
Spent mobile phone cells (LCO, 2.04 kg)	Donated post-consumer waste	N/A
Shredder (custom, <10 mm output)	Custom-fabricated, Suranaree University of Technology	N/A
Zigzag air classifier (14–35 CMM)	Custom-fabricated, Suranaree University of Technology	N/A
Circular screen vibrator (35-mesh/500 μm)	Custom-fabricated, Suranaree University of Technology	N/A
ICP-OES spectrometer (Optima 8000)	PerkinElmer	N/A

### Experimental model and study participant details

Omitted as our study does not involve biological models.

### Method details

#### Materials

The raw materials for this study were a spent electric vehicle (EV) battery module (state of health <60%), weighing approximately 13 kg (comprising 16 cells), and 2 kg of mobile phone (MP) batteries (collected mass) collected from local repair stores in Thailand. The technical specifications for both are detailed in Table S1. The chemical used for mobile phone battery discharging was an aqueous 10 wt % NaCl solution of refined salt (sodium chloride (NaCl), TRS^R^, 97.5%).

#### Battery discharging

Before the black mass separation process, the electric car and mobile phone batteries were subjected to a thorough and distinct discharge process. The electric car battery module was discharged to a voltage below 0.1 V using a custom-designed discharger by adjusting the resistance value with a series of resistors (Figure S1). Subsequently, the module was disassembled by removing the outer casing, plastic components, and electrical wiring. In contrast, the mobile phone batteries were discharged in a 10 wt % NaCl aqueous solution (1 kg of the batteries per 1 L of the NaCl solution) for 24 h, ensuring complete discharge, followed by a thorough water rinse and air drying at ca. 30 °C. This treatment step aims to avoid potential danger issues such as short-circuit, fire, and explosion during shredding in the mechanical size reduction dismantling step.^107^^,^^118^

#### Baseline black mass composition

To establish a baseline for evaluating the efficiency of the automated mechanical separation process, the weight composition of the cathode and anode active materials (black mass) was determined via manual dismantling after the batteries were fully discharged. This composition analysis serves as a control value representing the total recoverable black mass content. To ensure accuracy, the data collection was performed in triplicate, analysing three separate EV battery modules and three individual mobile phone cells. The final reported black mass compositions were calculated from the average weights recovered from these triplicate samples for both battery types. Figure 1A shows a manually disassembled EV battery module (total weight before disassembling ca. 13 kg), revealing 16 battery cells (ca. 11,680 g) and other components. One cell (ca. 730 g) was further dismantled to show its internal structure. The cell comprised anodes (ca. 218 g), cathodes (ca. 276 g), separators (ca. 53 g), and other structural parts. The active material (black mass) content within the EV battery module was quantified via a two-step physical separation method. First, the anode and cathode electrodes were subjected to air exposure for 1 h. Second, the active material layers were physically removed from the current collectors. The mass of the active material was indirectly measured by determining the difference between the initial electrode mass and the mass of the pure metallic current collector remaining after detachment. Remarkably, the anode active material layers on the copper electrode exhibited a propensity to delaminate spontaneously when exposed to ambient air.^118^ Within 5 min, the material layers began to crack and delaminate in a flake-like form. To ensure complete detachment, the electrodes were allowed to stand for an hour before the anode active materials were physically separated from the copper foil through gentle agitation.

In contrast, a disassembled mobile phone battery cell produced ca. 298 g of anode electrode, ca. 401 g of cathode electrode, and other components (Figure 1B). While the anode electrode is successfully separated using the same method as the EV battery, the mobile phone cathode electrode proved more challenging due to differences in discharging methods and chemical composition (see image of uncleaned mobile phone cathode delamination by leaving it in air, followed by a physical scratch in Figure S2). Therefore, heating at 500°C for 1 h is necessary to pyrolyse the binder before physical separation, yielding an active cathode material weight of ca. 357 g. Typically, polyvinylidene fluoride (PVDF) is used as the binder for the cathode, but polytetrafluoroethylene (PTFE) has also been used for the fluoride-based binder.^35^ The high-temperature treatment allows the decomposition of the binder and the evaporated organic materials embedded in the active material (such as residual electrolytes). Additionally, part of LiCoO_2_ (mixed with anode carbon materials) could be transformed into Co_3_O_4_ at 500°C and then transformed to CoO and Li_2_CO_3_ at 607°C–689°C.^119^^,^^120^ Similarly, the Co_3_O_4_ compound is found after the treatment of cathode materials at 500°C in the air in this study, as shown in Figure S3. Therefore, the weight of the black mass is calculated by the mass of the initial electrode minus the mass of the delaminated current collector plate (Cu and Al foil) to avoid mass loss of the black mass during the delamination experiment and mass changing of the mobile phone active cathode materials after the pyrolysis process.

The weight percentages of the battery components are calculated and presented in Figures 1C and 1D. For the EV battery, the weight of each cell’s components is multiplied by 16 to represent the entire module. The total weight percentages of the active anode and cathode materials are 17.35 (±2.14) wt. % and 29.98 (±0.20) wt. % for the EV battery module, while for the mobile phone cell, they are 25.83 (±0.14) wt. % and 45.33 (±1.11) wt. %, respectively. The total weight of the active materials (47.33 wt. % for the EV battery module and 71.16 wt. % for the mobile phone cell) obtained from manual dismantling, shown in Table S2, is used below to evaluate the efficiency of mechanical automation of black mass separation.

#### Black mass mechanical separation

The pre-discharged electric vehicle battery module (with the external casing removed) and mobile phone batteries were immediately processed in a custom-designed separation unit comprising a shredder, a zigzag air separator, and circular screen vibrator machines (Figure 2). This unit was designed by the Innovative Processing and Recycling of Metals Research Center at Suranaree University of Technology, Thailand. To ensure the accuracy and reproducibility of subsequent calculations, this mechanical process was systematically repeated three times for both battery types, utilising approximately 13 kg of EV battery module per run and approximately 2 kg per batch for the mobile phone batteries. The data for the separation calculations were collected by weighing the mass recovered from each component unit of the machine after every run.

The shredding machine (360 V) had a capacity of 100 kg h^−1^, with a shredder size (hammer mill) of 200 mm × 200 mm x 300 mm, controlled by a 7.5 kW motor (Figure 2A). This machine could cut materials to a size smaller than 10 mm and was connected to a nitrogen gas feed from the bottom to prevent undesirable reactions (25 L min^−1^ from a 47 L tank with 150 bar of N_2_). The sufficiently shredded materials were screened with a 10 mm diameter screen sieve installed under the shredder unit. Additionally, a dust and gas collector was installed on top of the shredding machine unit to capture particulate matter and volatile byproducts generated by the procedure.

The zigzag machine (Figure 2B) was used to separate the shredded materials, which consisted of cathode and anode materials, aluminum foil, copper foil, separator, and other plastic parts with varying densities. The zigzag air classifier was powered by a 0.75 kW blower motor controlled via a variable frequency drive (VFD), with a manufacturer-rated flow capacity of 14–35 CMM (m^3^ min^−1^). The VFD was operated at 23 Hz for the initial classification pass, and 55 Hz for the subsequent pass, spanning the blower’s rated range; airflow was not independently metered at each setting. Taking the full rated flow range across the 0.125 m inlet air damper (A = 0.0123 m^2^) gives an indicative superficial gas velocity range of approximately 19–47 m s^−1^ across the blower’s operating range. The materials from the top were controlled by a 0.37 kW feeder motor. The heavier materials were dropped to the bottom, while the lighter materials were sucked up by a suction pipe connected to a vacuum pump section.

The materials, either directly obtained from the shredding machine or processed through both the shredding and zigzag machines, were subjected to separation of the black mass using circular screen vibrator machines (Figure 2C). The circular screen vibrator machine, operating at 380 V with a three-phase power supply, was equipped with a 518 mm diameter sieve tank and vibration motor power of 30 W. A 35-mesh sieve (500 μm), which is sufficient for black mass screening,^121^^,^^122^ was installed to achieve processing that can operate with a 100 kg h^−1^ capacity.

The experimental investigation focused on the mechanical separation of black mass from spent lithium-ion batteries originating from two distinct sources: EV battery modules and mobile phone cells. To ensure experimental robustness, the separation procedure for each battery source was replicated three times. Spent EV battery modules underwent an initial two-stage separation process. After disassembly, the interconnected 16-cell assembly was processed via a sequential combination of shredding, zigzag air separation, and circular screen vibration. Two distinct processing routes were evaluated to optimise the black mass yield: Route EV I: Involved sequential shredding, initial circular screen vibration, zigzag air separation at two motor frequencies (23 Hz and 55 Hz), followed by a final circular screen vibration. Route EV II: Commenced with shredding and zigzag air separation at 23 Hz. This was followed by two sub-routes: one incorporating initial circular screen vibration for lighter materials, and the other involving zigzag air separation at 55 Hz for heavier materials, culminating in a final circular screen vibration. The final black mass product was collected by combining the output from the first and second passes of the final circular screen vibration stage in both routes.

For mobile phone cells, a 2 kg batch of brine-discharged cells was directly fed into the black mass separation unit. Two processing routes were again employed: Route MP I: Included sequential shredding, coarse screening, and two-stage air classification (23 Hz) using a zigzag air separator. The heavier fraction from the initial air classification proceeded to a second round of air classification (55 Hz), followed by a second screening pass. The lighter fraction underwent only the second screening pass. Route MP II: Followed a similar sequence to Route MP I, but the order of the initial air classification and screening steps was reversed. Consistent with the EV approach, the black mass product was recovered from the final stages of both routes by combining the output from the first and second passes of the circular screen vibration.

#### Techno-economic and life cycle assessment

Data for the techno-economic analysis were determined by quantifying the mass of the separated products (black mass, plastic separator, cell case, copper, and aluminum foils) obtained from the optimal route identified for each battery type (EV and MP). Input costs, including electricity, chemical usage and waste disposal, were calculated based on prevailing industrial prices in Thailand. Revenue from separated products was determined using generic market prices, normalised per kilogram of material input.

The life cycle assessment shares the same process inventory as the techno-economic analysis for electricity, chemical reagent, and waste-solution flows, but adopts a distinct end-of-life boundary for the cell casing, wiring, and copper/aluminum foil streams: the techno-economic analysis models these as sold products at prevailing scrap prices, while the life cycle assessment conservatively assumes incineration, since downstream reuse of these streams cannot be guaranteed across all operating contexts. The environmental materiality of this boundary choice is quantified in the life cycle analysis section. The life cycle assessment shared the same scope and functional unit as the techno-economic analysis: the treatment and separation of 1 kg of incoming battery waste into its constituent components to produce a mixed black mass. Environmental impact per module was also reported for contextual reference. The primary environmental metric quantified was the Global Warming Potential (GWP), calculated using the IPCC 2021 methodology. Supplementary metrics assessed included water toxicity, land usage, and water scarcity, employing methodologies outlined by the European Union and weighted according to their proximity to EU 2020 policy targets. The EU-centric methodologies were selected for their real-world policy applicability, as comparable Asia-based frameworks were unavailable in the utilised software. Both process routes (EV and MP) were modeled and assessed using Umberto 11 software in conjunction with the Ecoinvent 3.11 database. This database supplied secondary data for inputs such as solvent production, electricity generation, and waste treatment. Electricity usage was specifically modeled based on the 2022 electricity production mix in Thailand.

Black mass reference prices (NMC622, LCO) were established using two independent methods, cross-validated against one another. The first method used single-day market price assessments for black mass (metal.com, accessed 31 July–3 August 2026).^85^^,^^86^ The second method calculated a payable-weighted price from contained-metal reference prices: nickel (LME cash official price),^84^ cobalt (Trading Economics and Intratec assessments),^87^^,^^88^ and battery-grade lithium carbonate (Shanghai Metals Market),^123^ each averaged across two to three dated points spanning December 2025 to August 2026. Lithium carbonate prices were converted to an elemental-lithium-equivalent basis using Li_2_CO_3_ stoichiometry (Li_2_CO_3_ molar mass 73.89 g/mol; 2 × Li = 13.88 g/mol; elemental Li fraction = 18.78%). The resulting per-kilogram metal values were weighted by the manufacturer-stated metal content of each product (NMC622: 31% Ni, 10% Co, 6% Li; LCO: 27.5% Co, 3.25% Li, midpoints of manufacturer-specified ranges) and scaled by an 80% payable rate, consistent with documented industry payable percentages of 70–85% for nickel-cobalt-manganese black mass, which reached a record 87.5% (exceeding 100% for high-purity material) in January 2026 amid strong Asian demand.^90^ The two methods agreed within 3% for NMC622 ($14.43/kg direct assessment vs. $14.01/kg payable-weighted) and within 17% for LCO ($12.97/kg vs. $15.14/kg), the latter divergence attributed to the comparatively thin and volatile nature of the LCO black mass market, evidenced by its markedly wider single-day trading spread (22.7%) relative to NMC622 (2.6%). The average of both methods—$14.22/kg for NMC622 and $14.05/kg for LCO—was adopted as the reference price for all subsequent calculations.

Given the documented volatility in battery raw material prices, a sensitivity analysis was performed to assess the robustness of the techno-economic conclusions, rather than relying on a static price assumption. Five variables were considered: black mass/contained-metal price (incorporating both underlying metal price and payable percentage, combined into a single multiplicative price-realisation factor for tractability), feedstock acquisition cost, electricity price, plant utilisation, and separation efficiency. The price-realisation factor was sampled from a triangular distribution (0.55–1.35× the base-case price, mode 1.0×), reflecting the documented 2022–2026 range of lithium, cobalt, and nickel price movements—including an approximately 85% peak-to-trough decline in lithium carbonate between 2022 and 2025,^124^ a subsequent recovery exceeding 100% from that trough by early 2026,^125^ and an approximately 160% cobalt price rally from its February 2025 low^89^—and the observed 70–85% range of black mass payable percentages.^90^ Electricity price was varied ±15% (triangular distribution), reflecting typical industrial tariff variation. Plant utilisation was sampled from a triangular distribution (50–100% of the 200,000 kg/yr design throughput underlying the overhead calculation, Table S6), reflecting pilot-to-commercial ramp-up risk. Separation efficiency was sampled from a normal distribution using the measured mean and standard deviation reported in Table 1 for each route. Feedstock cost was sampled from a triangular distribution spanning −$3.00/kg (a favourable gate-fee arrangement) to +$5.00/kg (a high-competition purchase price informed by China EXW battery-scrap assessments of 29,800–32,000 yuan/tonne, approximately $4.15–4.45/kg),^126^ with a mode at the base-case purchase price (Scenario B). One-at-a-time (tornado) and combined (Monte Carlo, *N* = 100,000) analyses were performed for both routes using Python 3 (NumPy).

### Quantification and statistical analysis

Separation efficiencies, recovered mass fractions, and elemental compositions are reported as mean ± standard deviation (SD) across *n* = 3 independent batch separation runs per route; dispersion measures are reported in Table 1 and the corresponding figure legends. No statistical hypothesis tests were performed; uncertainty in the techno-economic conclusions was quantified by one-at-a-time (tornado) and Monte Carlo (*N* = 100,000 draws, fixed random seed) sensitivity analyses implemented in Python 3 (NumPy), with input distributions described in the method details and results reported in the Techno-economic assessment section and Figures S7 and S8. No data were excluded from the analyses.
