# Supplementary material for: Baseline techno-economic and life cycle assessment of black mass production
Source: iScience. 2026 Oct 1;29(10):117729. doi: 10.1016/j.isci.2026.117729 (PMC13657251; doi:10.1016/j.isci.2026.117729)
Supplement: Supplementary file 1 [file mmc1.pdf]

iScience, Volume *29*

Supplemental information

Baseline techno-economic and life cycle assessment of black mass production

Tanongsak Yingnakorn, Chatisa Kansomket, Chaiyasit Longbutsri, Sean Scott, Tapany Patcharawit, Jake M. Yang, Andrew P. Abbott, and Sakhob Khumkoa

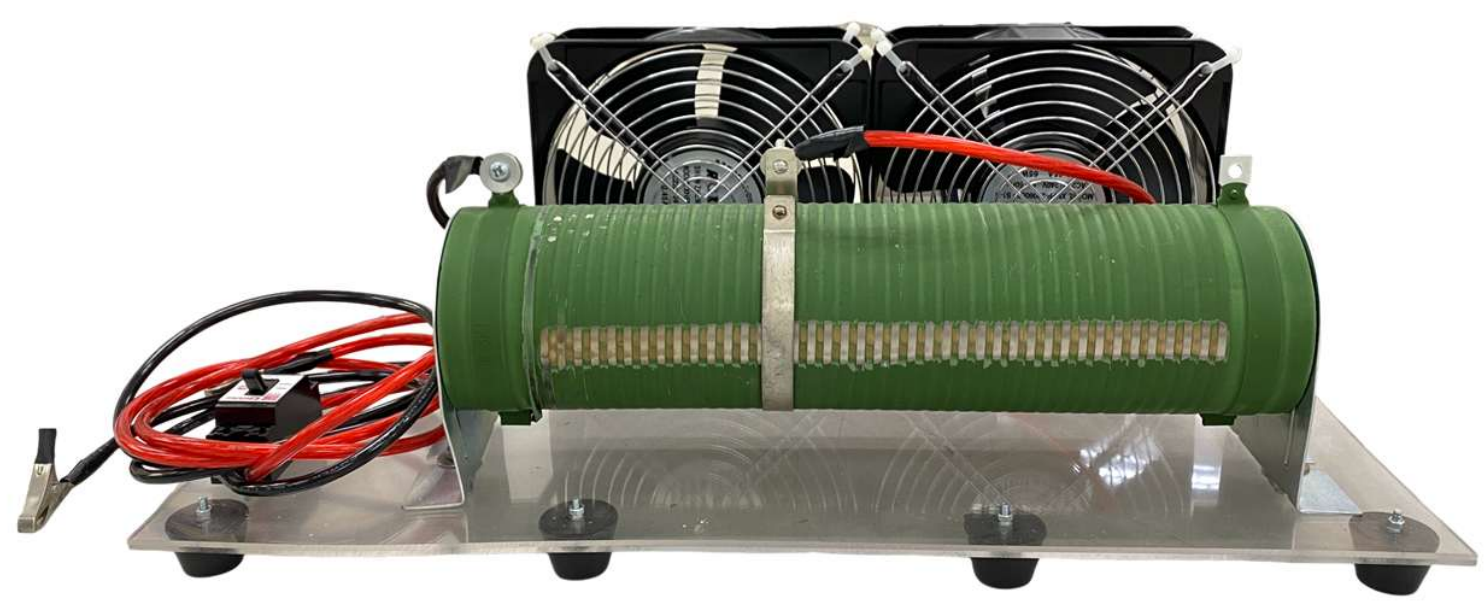

Figure S1. Custom-designed discharger device for EV battery modules, related to STAR Methods.

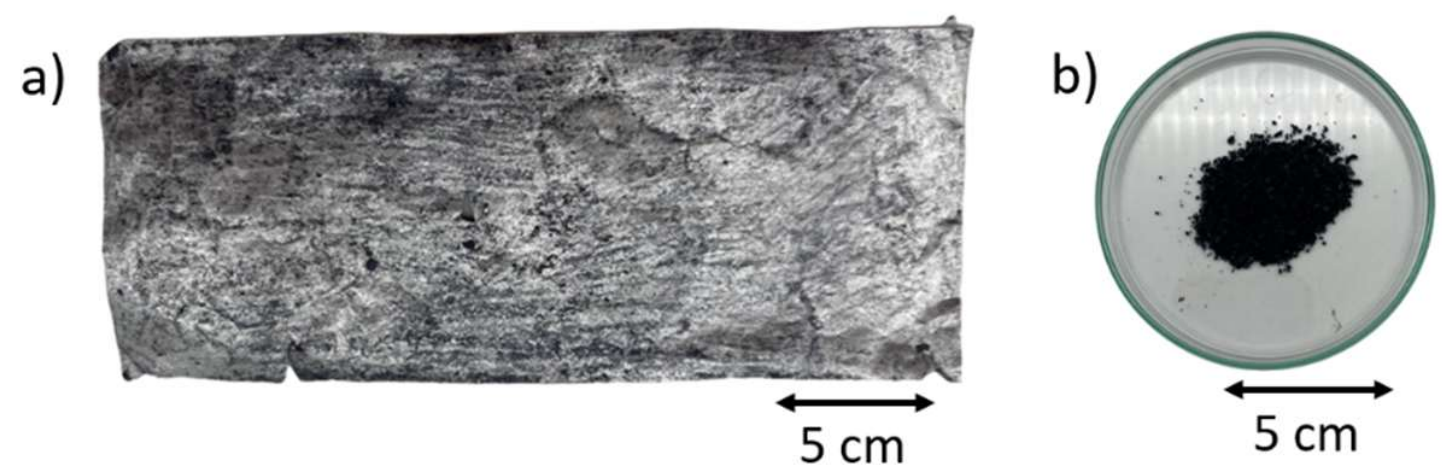

Figure S2. Images of a) an uncleaned mobile phone cathode, and b) the black mass after delamination by leaving in air, followed by physical scratch, related to STAR Methods. Scale bars: 5 cm.

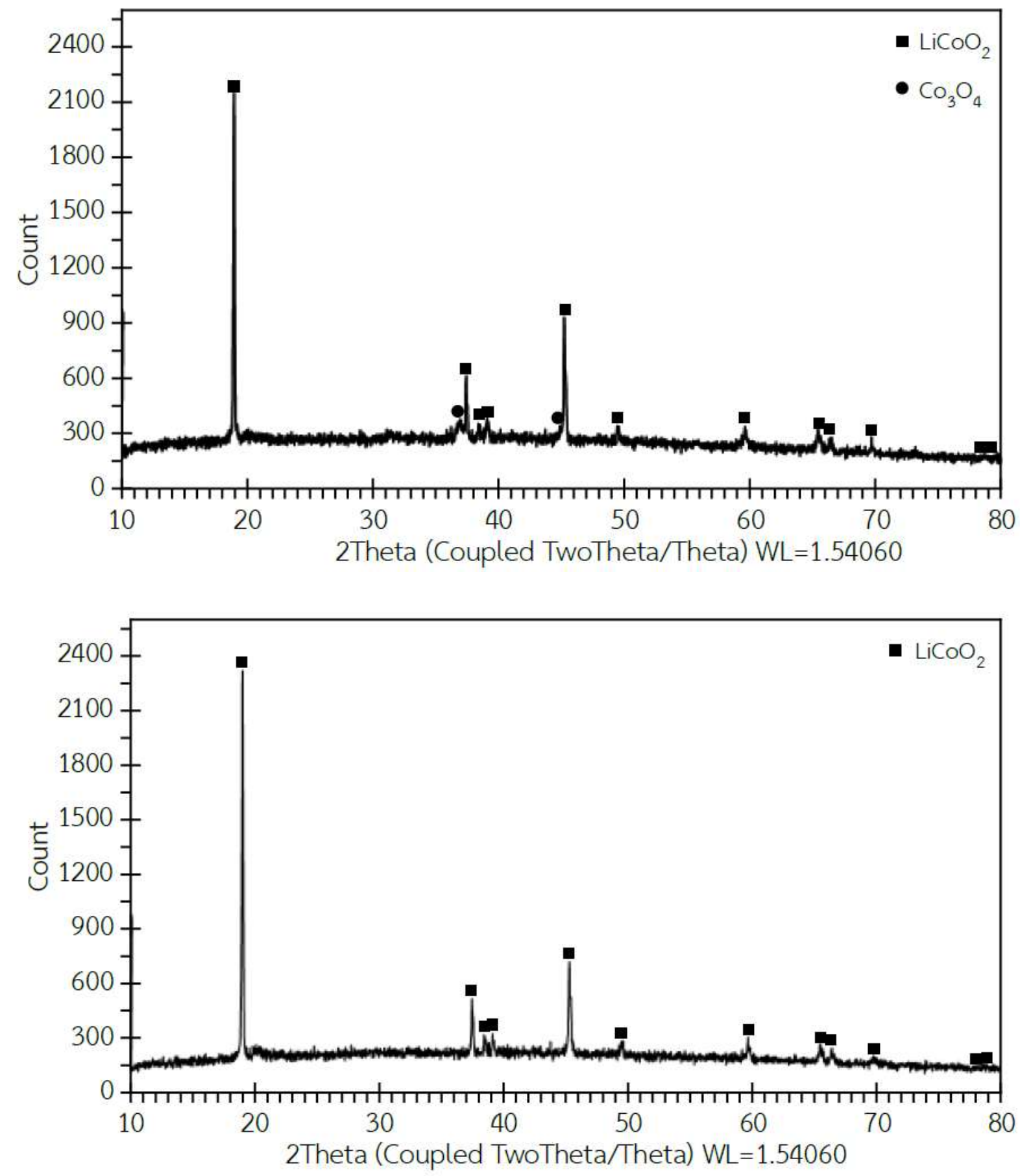

Figure S3. XRD patterns of initial spent mobile phone cathode materials (top) and after treatment (below) at 500°C for 1 h, related to Figure 3.

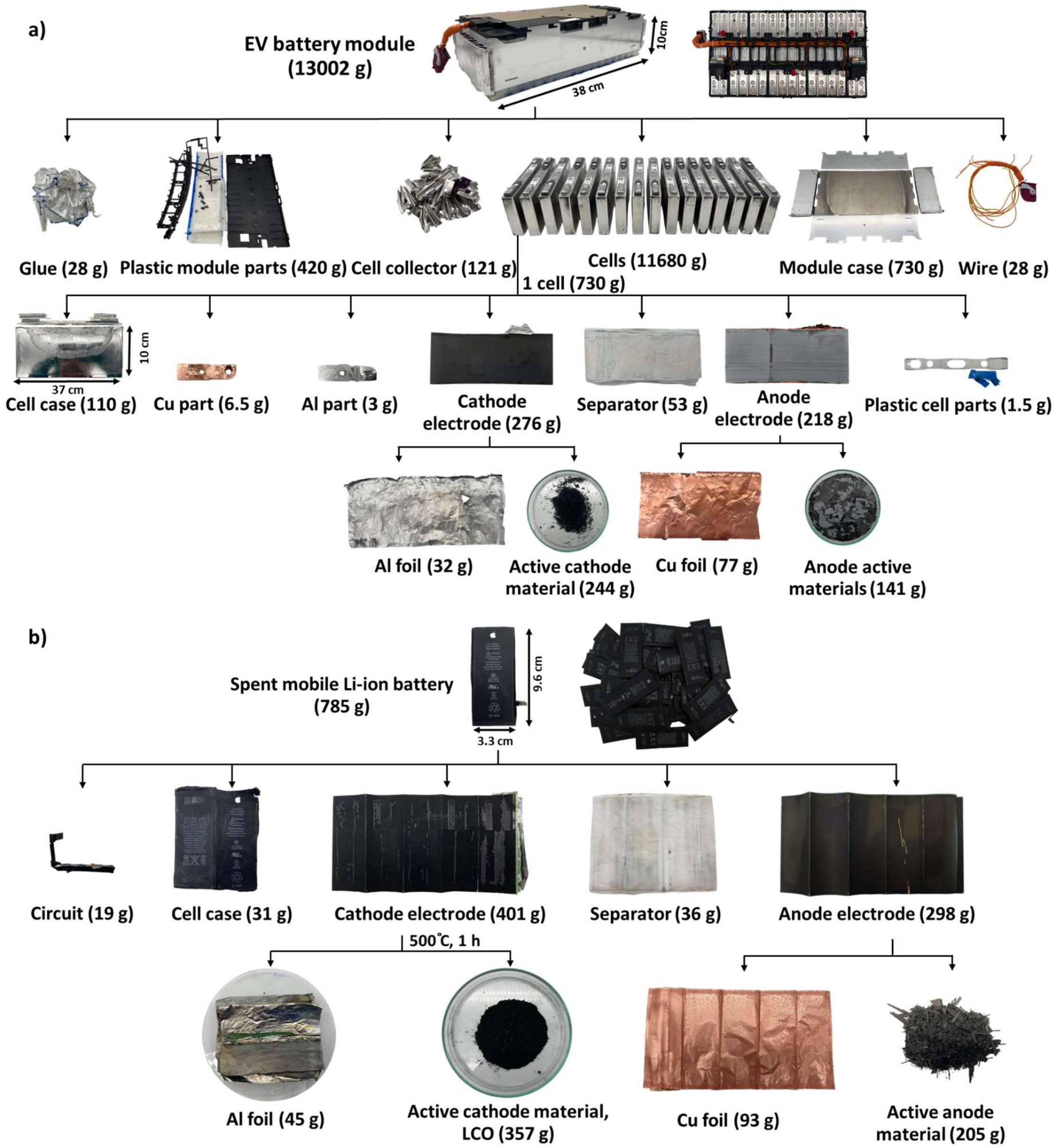

Figure S4. Components and their average weight of a) the electric vehicle and b) mobile phone batteries used in this study, related to Figure 1.

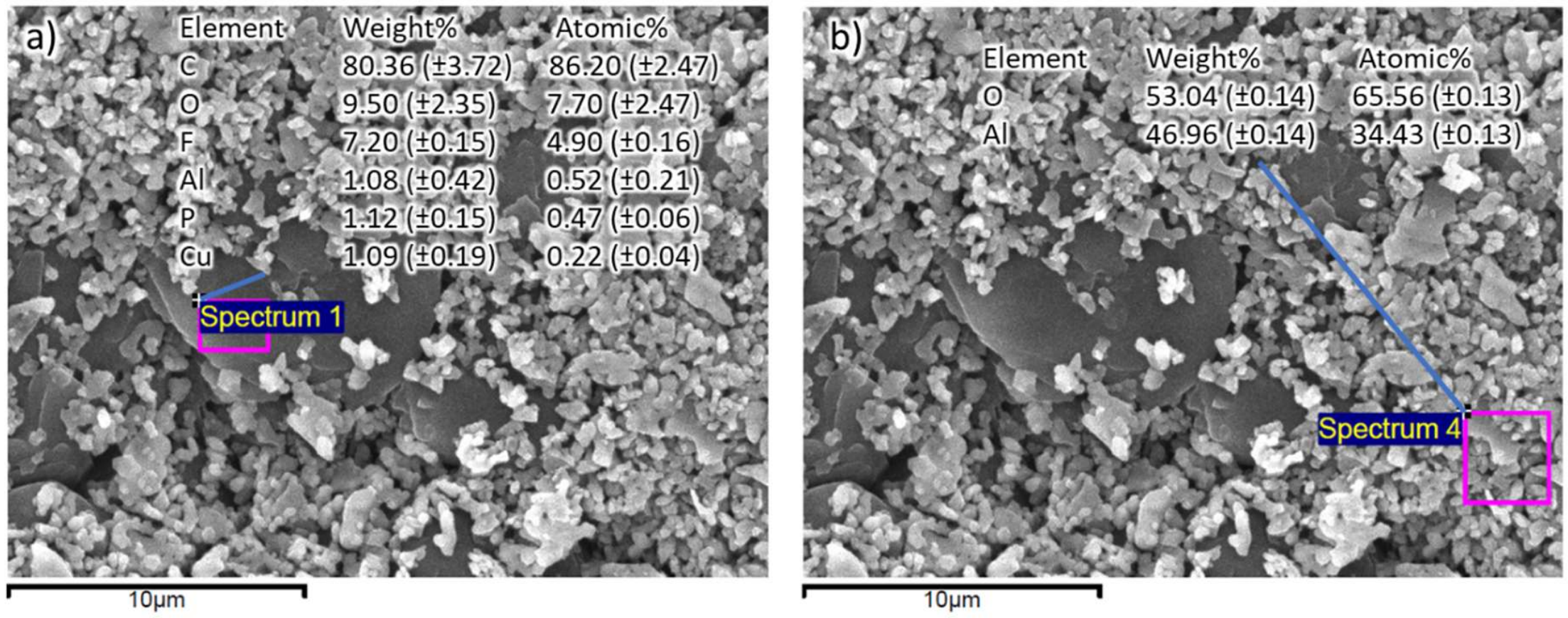

Figure S5. SEM and EDS results of spent EV anode surface: chemical composition of a) graphite areas and b) Al_2O_3 coating areas, related to Figure 3. Scale bars: 10 μm. Data are represented as mean (±SD).

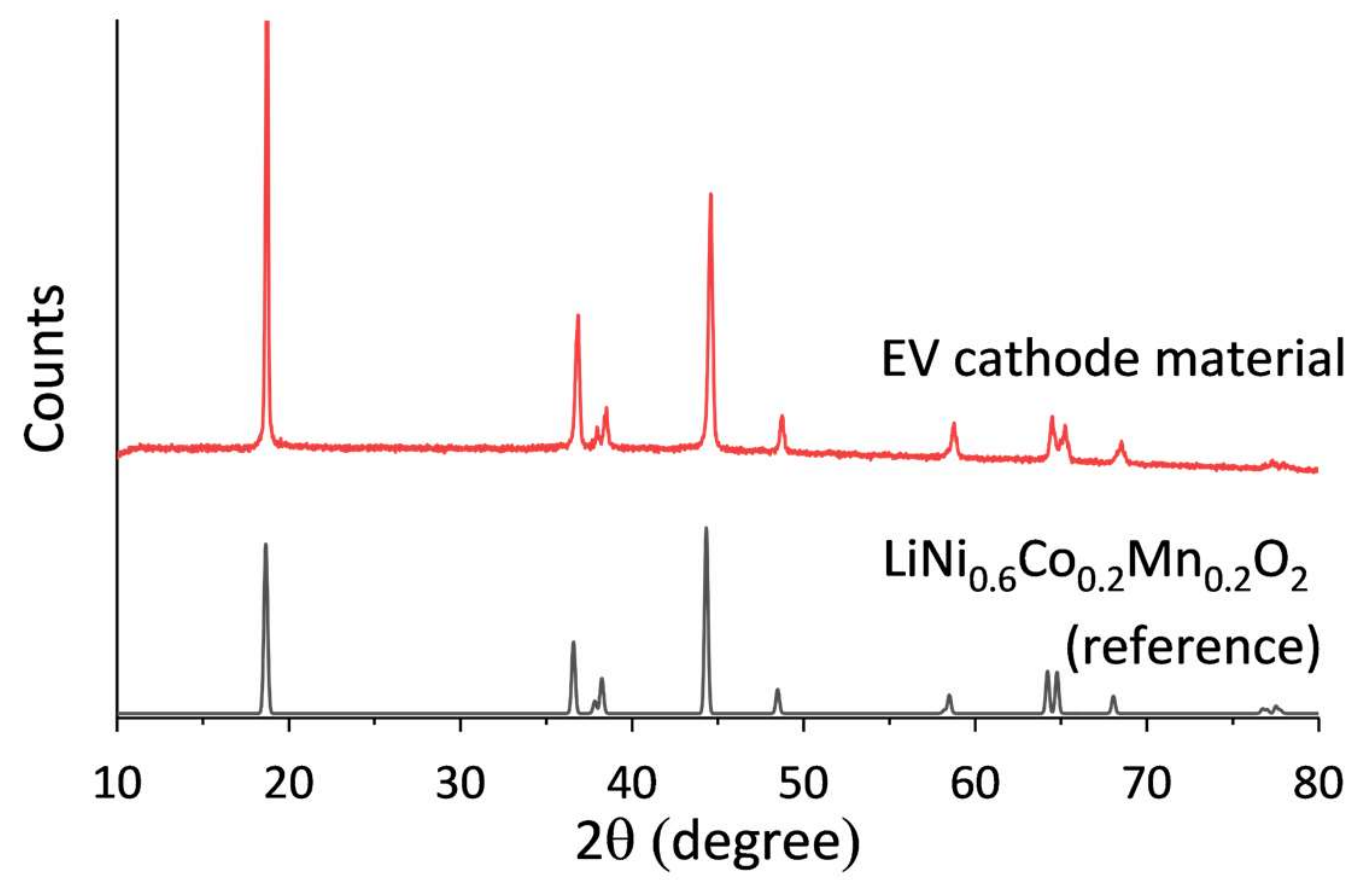

Figure S6. XRD pattern of spent EV active cathode materials, related to Figure 3.

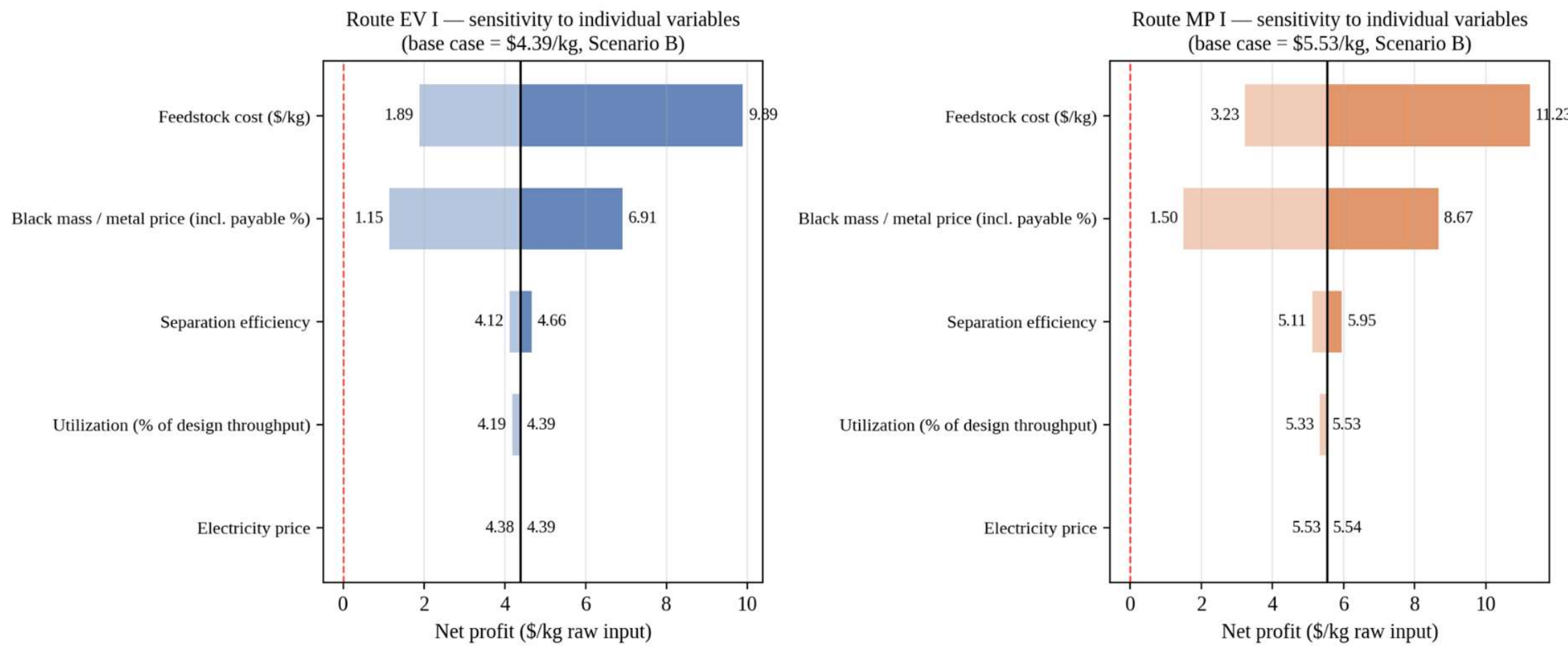

Figure S7. Tornado sensitivity analysis of net profit (Scenario B, feedstock purchase basis) to five key techno-economic variables for Route EV I and Route MP I, showing the profit range resulting from varying each variable individually across its stated bounds while holding all others at base case. Feedstock cost and black mass/contained-metal price are the dominant drivers for both routes, related to Table 1.

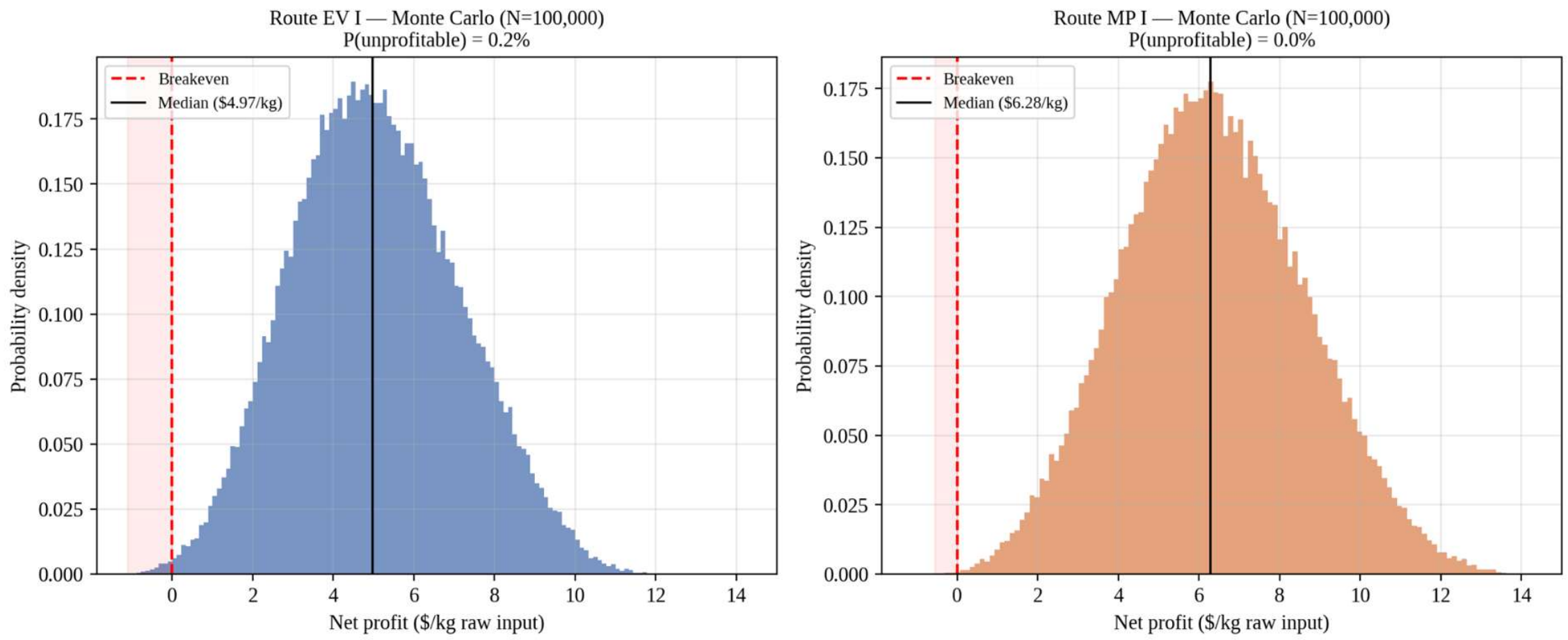

Figure S8. Monte Carlo simulation (N = 100,000) of net profit under combined, simultaneous uncertainty across five key techno-economic variables for Route EV I and Route MP I. The red dashed line marks the breakeven point (zero profit); the shaded region indicates the unprofitable range. Median and probability of unprofitability are annotated for each route, related to Table 1.

Table S1. Technical specifications of the spent batteries, related to STAR Methods.

**Parameter**	**EV Battery Modules**	**MP Batteries**
Chemistry	Lithium-ion (NMC)	Lithium-ion (LCO)
Total Mass Evaluated	~13 kg (16 cells)	2 kg (collected bulk)
State of Health (SOH)	< 60%	Not specified
Nominal Voltage	59.2 V (3.7 V per cell)	3.8 V (per cell)
Capacity	25–26 Ah	1440–1570 mAh (per cell)
Nominal Energy	~1.5 kWh	5.47–5.92 Wh (per cell)

Note: Because these materials were obtained as bulk post-consumer waste donations and collections from local repair stores, their historical operation profiles, service durations, and cycling histories are untraceable. The precise manufacturer and model designations cannot be disclosed due to commercial sensitivity, which is not important to this study.

Table S2. Components of the dismantled EV battery module and mobile phone cell, related to Figure 1.

<table>
<tr><th>Source</th><th colspan="3">Component</th><th>wt%</th></tr>
<tr><td rowspan="16">EV battery module
(Total weight of 13,002.33 (±4.04) g)</td><td rowspan="10">Battery cells (16 cells,
total weight of 11,676.26(±3.91) g)</td><td rowspan="2">Anode electrodes</td><td>Cu foil</td><td>9.52 (±1.87)</td></tr>
<tr><td>Active anode materials</td><td>17.35 (±2.14)</td></tr>
<tr><td rowspan="2">Cathode electrodes</td><td>Al foil</td><td>3.95 (±0.48)</td></tr>
<tr><td>Active cathode materials</td><td>29.98 (±0.20)</td></tr>
<tr><td colspan="2">Cell case</td><td>13.57 (±0.38)</td></tr>
<tr><td colspan="2">Cu part</td><td>0.80 (±0.01)</td></tr>
<tr><td colspan="2">Al part</td><td>0.37 (±0.01)</td></tr>
<tr><td colspan="2">Plastic parts</td><td>0.18 (±0.00)</td></tr>
<tr><td colspan="2">Separator</td><td>6.57 (±0.46)</td></tr>
<tr><td colspan="2">Other</td><td>7.50 (±0.76)</td></tr>
<tr><td colspan="3">Module case</td><td>5.61 (±0.00)</td></tr>
<tr><td colspan="3">Plastic parts</td><td>3.23 (±0.01)</td></tr>
<tr><td colspan="3">Cell connectors</td><td>0.93 (±0.01)</td></tr>
<tr><td colspan="3">Glue plates</td><td>0.21 (±0.00)</td></tr>
<tr><td colspan="3">Wires</td><td>0.22 (±0.00)</td></tr>
<tr><td colspan="3">Total</td><td>100</td></tr>
<tr><td rowspan="8">Mobile phone battery cell
(Total weight of 784.52 (±5.52) g)</td><td colspan="2" rowspan="2">Anode electrode</td><td>Cu foil</td><td>12.05 (±1.26)</td></tr>
<tr><td>Active anode materials</td><td>25.83 (±5.62)</td></tr>
<tr><td colspan="2" rowspan="2">Cathode electrode</td><td>Al foil</td><td>5.82 (±0.07)</td></tr>
<tr><td>Active cathode materials</td><td>45.33 (±2.76)</td></tr>
<tr><td colspan="3">Cell case</td><td>3.97 (±0.57)</td></tr>
<tr><td colspan="3">Circuit</td><td>2.42 (±0.01)</td></tr>
<tr><td colspan="3">Separator</td><td>4.59 (±0.78)</td></tr>
<tr><td colspan="3">Total</td><td>100</td></tr>
</table>

Note: Total active material content (anode + cathode active materials combined) is 47.33 wt% for the EV battery module (17.35% + 29.98%) and 71.16 wt% for the mobile phone cell (25.83% + 45.33%), as referenced in the main text. Data are represented as mean (±SD); n = 3 independent batch separation runs.

Table S3. Cost estimate for 1 kg of EV battery module raw material, related to Table 1.

Category	Item	Quantity	Units	Cost ($)	Revenue ($)	Profit ($)
Raw material	Battery module	1.0000	kg	-	-	
Process cost	Electricity*	0.2360	kW h	0.0309	-	
	N_2 gas (99.9%)	13.6385	L	0.0754	-	
Product	Plastic module parts	0.0323	kg	-	0.0039	
	Module case	0.0562	kg	-	0.0991	
	Wire	0.0023	kg	-	0.0032	
	Black mass	0.4415	kg	-	3.3778	
	Separator	0.1415	kg	-	0.0129	
	Cu and Al foils	0.1538	kg	-	0.6555	
	Cell case	0.1169	kg	-	0.1423	
Sum				0.1063	4.2947	4.1885

Table S4. Cost estimate for 1 kg of mobile phone battery raw material, related to Table 1.

Category	Item	Quantity	Unit	Cost ($)	Revenue ($)	Profit ($)
Raw material	Battery cells	1.0000	kg	-	-	
Process cost	Electricity*	0.2710	kW h	0.0355	-	
	N_2 gas (99.9%)	15.0000	L	0.0829	-	
	NaCl salt	0.1000	kg	0.0664	-	
	Water	0.9000	L	0.0411	-	
	Waste disposal	1.0000	L	0.3043		
Product	Black mass	0.5400	kg	-	3.6990	
	Separator	0.0500	kg	-	0.0046	
	Cu and Al foils	0.3200	kg	-	1.3634	
	Cell case	0.0100	kg	-	0.0122	
Sum				0.5302	5.0791	4.5489

Table S5. Electricity used in this study of Route EV I and MP I per 1 kg of the raw material. These data were calculated from the maximum capacity (100 kg/hr) of the machines and the flow rate of 14-35 CMM for the zig-zag separating machine, related to Tables 1 and 2.

**Process**	**Step**	**Electricity (kWh)**
Route EV I	Shredding	0.088
	Screen Vibration	0.003
	Zig-zag Separation (23 Hz)	0.086
	Zig-zag Separation (55 Hz)	0.058
	Screen Vibration	0.001
**Total**		**0.236**
Route MP I	Shredding	0.099
	Screen Vibration	0.004
	Zig-zag Separation (23 Hz)	0.091
	Screen Vibration	0.001
	Zig-zag Separation (55 Hz)	0.076
	Screen Vibration	0.002
**Total**		**0.271**

Methods S1. Derivation and mathematical modelling of the industrial fixed overhead factor ($0.20/kg), related to Table 1 and Table S6.

To scale up the raw direct material and energy inventory costs recorded at the laboratory scale to a realistic "factory-gate" economic baseline, a customised fixed-overhead framework was established. The scale-up model is structurally tied to the pilot-scale processing rate of the customised mechanical-aerodynamic flowsheet, which operates at a baseline design capacity of **100 kg/hr** (as detailed in **Table S4**). Assuming standard industrial single-shift parameters—comprising 8 operating hours per day and 250 operational days per calendar year—the annual processed mass throughput (M_{annual}) for the pilot plant is defined as:

$$M_{annual} = 100\ \text{kg/hr} \times 8\ \text{hours/day} \times 250\ \text{days/year} = 200{,}000\ \text{kg/year}$$

The total fixed industrial overhead factor ($C_{overhead}$ = $0.20/kg) is mathematically partitioned across three localised corporate categories:

1. Equipment & Infrastructure Capital Depreciation ($C_{depreciation}$ = $0.05/kg)
The initial capital expenditure (CAPEX) for the core pilot sorting assembly is estimated at approximately $100,000 USD. Following standard plant accounting heuristics, a 10-year straight-line depreciation matrix with zero salvage value is applied, yielding a fixed annual depreciation expense of $10,000 USD. The normalised depreciation cost per kilogram of battery feedstock is calculated as:

$$C_{depreciation} = \frac{\$10{,}000\ /year}{200{,}000\ kg/year} = \$0.05/\ \text{kg}$$

2. Localised Direct Manufacturing Labour (C_{labor} = $0.06/kg)
Continuous operation of the mechanical sorting line requires a steady-state labour allocation of two full-time technicians per shift. Drawing from localised industrial manufacturing wage structures in Thailand, the gross labour cost per technician is modelled at 21,000 THB/month (inclusive of base salary, social security, and allowances). At the specified exchange rate baseline of **32.86 THB/USD**, this corresponds to **$639.07 USD/month** per operator. The gross annual direct labour requirement is defined as:

$$\text{Annual Labor Expense} = 2\ \text{operators} \times \$639.07/\text{month} \times 12\ \text{months/year} = \$15{,}337.68/\text{year}$$

$$\text{Unoptimised Labour Cost Component} = \frac{\$15{,}337.68/year}{200{,}000\ kg/year} = \$0.077/\ \text{kg}$$

Assuming typical multi-line manufacturing plant optimisation and shared facility integration efficiencies, this metric is allocated at a baseline cost factor of **$0.06 / kg**.

3. Plant Operational Management & Facility Overhead ($C_{management}$ = $0.09/kg)
Following established economic guidelines for plant design, factory-gate overhead accounts for indirect operational charges (facility leasing fees, property insurance, environmental compliance monitoring, safety permits, maintenance parts, and a standard 15% plant management overhead multiplier). Aggregated for a localised pilot plant situated in a Thai industrial zone, this equates to an annual budget of approximately $18,000 USD:

$$C_{management} = \frac{\$18{,}000/year}{200{,}000\ kg/year} = \$0.09/\text{kg}$$

Table S6. Itemised breakdown of the fixed industrial overhead cost model, related to Table 1.

Cost Pillar	Base Financial Inventory Parameter	Mass Throughput Basis	Calculated Cost Component
Capital Infrastructure Depreciation	$100,000 CAPEX (10-Year Straight-Line Matrix)	200,000 kg/year	$0.05 / kg
Localised Manual Labour	2 Operators @ 21,000 THB/month gross (~$639.07 USD)	200,000 kg/year	$0.06 / kg
Plant Management & Facility Overhead	Industrial lease, compliance, 15% management fee	200,000 kg/year	$0.09 / kg
Total Aggregated Fixed Overhead ($C_{Overhead}$)	—	—	$0.20 / kg

Methods S2. Definitions of life cycle impact categories and additional modelling assumptions, related to Table 3 and Tables S7–S9.

This section provides definitions and additional data necessary to complete the life cycle analysis shown in the main text. The definitions for the key environmental impacts discussed are: Global Warming Potential – an Indicator of potential warming effects from greenhouse gas emissions, scaled relative to carbon dioxide equivalents. Usually defined as the warming effect that occurs over the course of 100 years. Eco-toxicity (water) – Impacts on water-based organisms from toxic substances emitted into the environment (measured in CTUe on a 1-100 scale). Land use – dimensionless quantity relating to changes occurring within the land as a result of the process, namely changes to soil quality through biotic production, erosion and mechanical filtration (measured in 'points', which is an aggregated index of kg biotic production/(m^2*a) and kg soil (m^2*a)). Water scarcity – Indicates the relative water usage scaled to the region's water scarcity factors, measured by the m^3 of water deprived from the region due to the process (m^3 world equiv.). Table S4 shows the key datasets obtained from the Ecoinvent 3.11 database that were used for the life cycle analysis data given in Table 2.

Table S7. A list of processes obtained from the Ecoinvent 3.11 database to account for solvent production, electricity generation, and waste treatments, related to Table 3.

Dataset needed	Unit in the database process	Source from Ecoinvent 3.11	Assumptions
Electricity	1 kWh (Split into Thailand grid mix; 56.4% - Gas, 19.6% - Coal, 9.8% - Biofuel, 5.7% - Oil, 3.7% - Hydro, 2.7% - Solar, 1.6% - Wind, 0.5% - Waste)	electricity production, natural gas, conventional power plant (ID),<br>electricity production, lignite (TH),<br>heat and power co-generation, biogas, gas engine (TH),<br>electricity production, oil (TH),<br>electricity production, hydro, reservoir, tropical region (TH)<br>electricity production, photovoltaic, 3KWp slanted-roof installation, single-Si panel, mounted (TH)<br>electricity production, wind, <1MW turbine, onshore (ID)<br>heat and power co-generation, wood chips, 6667 kW (TH)	Some sources relate to Indonesia instead of Thailand, as no Thailand-specific or 'rest of world' datasets were available.
Nitrogen gas	1 kg	argon to the generic market for inert gas for discharge lamps (GLO)	Argon-specific data needed to be used, as no nitrogen gas datasets were present.
Sodium chloride	1 kg	sodium chloride production, powder (RoW)	
Deionised water	1 kg	water production, deionised (RoW)	
Waste plastics	1 kg treated	waste plastic, mixture (GLO)	Assumed to be treating separators, glues and plastic module parts in waste scenarios.
Waste aluminium	1 kg treated	treatment of aluminium in car shredder residue, municipal incineration (GLO)	In most scenarios, this is assumed to be the treatment for metal components.
Waste wiring	1 kg treated	treatment of waste electric wiring, collection for final disposal (GLO)	
Waste solvent	1 kg treated	treatment of spent solvent mixture, hazardous waste incineration (RoW)	

Table S8. Life cycle inventory for the processes discussed in this study, Route EV I and MP I. Inputs/outputs for reagents, solvents and electricity are included, related to Table 3.

Process	Step	Input/output	Contributor	Quantity	Unit
Route EV I	Disassembly	Inputs	Incoming waste battery module	13	kg
		Outputs	Plastic Module Parts	0.42	kg
			Disassembled Module	11.82	kg
			Module Casing	0.73	kg
			Wire	0.03	kg
	Shredding	Inputs	Disassembled Module	11.82	kg
			Electricity	1.14	kWh
			N_2 Gas	177.3	L
		Outputs	Shredded Materials	11.3	kg
			Lost Material	0.52	kg
			N_2 Gas	177.3	L
	Screen Vibration	Inputs	Shredded materials	11.3	kg
			Electricity	0.0452	kWh
		Outputs	Large size Faction	5.64	kg
			Black Mass	5.52	kg
			Lost Material	0.14	kg
	Zig-zag Separation (23 Hz)	Inputs	Large size Faction	5.64	kg
			Electricity	1.11	kWh
		Outputs	Heavy Faction	3.8	kg
			Separator	1.84	kg
	Zig-zag Separation (55 Hz)	Inputs	Heavy Faction	3.8	kg
			Electricity	0.7504	kWh
		Outputs	Light Faction	2.22	kg
			Cell Casing	1.52	kg
			Lost Materials	0.06	kg
	Screen Vibration	Inputs	Light Faction	2.22	kg
			Electricity	0.00888	kWh
		Outputs	Cu and Al foils	2	kg
			Residual Black Mass	0.22	kg
Route MP I	Discharge	Inputs	MP Battery Module	2.04	kg
			NaCl	0.204	kg
			Water	1.836	L
		Outputs	Discharged Battery	2.04	kg
			Waste Solution	2.04	kg
	Shredding	Inputs	Discharged Battery	2.04	kg
			Electricity	0.194	kWh
			N_2 Gas	30.60	L
		Outputs	Shredded Materials	1.96	kg
			Lost Materials	0.08	kg
			N_2 Gas	30.60	L
	Screen Vibration	Inputs	Shredded Materials	1.96	kg
			Electricity	0.00784	kWh
		Outputs	Large Size Faction	0.9	kg
			Black Mass	0.98	kg
			Lost Material	0.08	kg
	Zig-zag Separation (23 Hz)	Inputs	Large Size Faction	0.9	kg
			Electricity	0.1776	kWh
		Outputs	Heavy Faction	0.75	kg

			Light Faction	0.14	kg
			Lost Material	0.01	kg
	Screen Vibration	Inputs	Light Faction	0.14	kg
			Electricity	0.00056	kWh
		Outputs	Separator	0.1	kg
			Residual Black Mass	0.04	kg
	Zig-zag Separation (55 Hz)	Inputs	Heavy Faction	0.75	kg
			Electricity	0.148	kWh
		Outputs	Cell Casing	0.02	kg
			Light Materials	0.73	kg
	Screen Vibration	Inputs	Light Materials	0.73	kg
			Electricity	0.00292	kWh
		Outputs	Cu and Al Foils	0.65	kg
			Residual Black Mass	0.08	kg

Table S9. Comparative LCA impact indicators for Route EV I under the baseline Thailand electricity grid vs. a low-carbon regional grid scenario, related to Table 3.

Impact Category	Unit	Baseline Grid (per kg Input)	Baseline Grid (per kg Black Mass)	Low-Carbon Grid (per kg Input)	Low-Carbon Grid (per kg Black Mass)	Reduction (%)
Global Warming Potential	kg CO_2 eq.	0.21	0.476	0.078	0.177	62.80%
Freshwater Ecotoxicity	CTUe	0.406	0.920	0.212	0.480	47.80%
Land Use	Points	0.169	0.383	0.085	0.193	49.70%
Water Scarcity	m^3 world eq.	0.027	0.061	0.009	0.020	66.70%

Table S10. Reported annual throughput of named commercial and pilot/demonstration black-mass or battery-pretreatment operations, related to Table 2.

Operation	Type	Reported throughput	Ref.
Fortum, Harjavalta, Finland	Commercial	~3,000 t/yr	S1
Northvolt Revolt, Sweden	Commercial	~25,000 t/yr	S2
BASF, Schwarzheide, Germany	Commercial	15,000 t/yr	S3
Li-Cycle Spokes (Kingston/Rochester/Gilbert/Tuscaloosa)	Commercial	5,000–10,000 t/yr each	S4
Li-Cycle Rochester Hub	Commercial	35,000 t/yr black mass processing	S4
Blue Whale Materials, Bartlesville, OK	Commercial	14,000 t/yr	S5
LG Energy Solution / Toyota Tsusho JV, Winston-Salem, NC	Commercial (from 2026)	13,500 t/yr	S2
Redwood Materials, Nevada	Commercial	60,000 t/yr	S6
Umicore (current European operations)	Commercial	~7,000 t/yr	S7
GEM Co., China	Commercial	300,000 t/yr	S2
CATL Brunp, China	Commercial	500,000+ t/yr	S2
Pyrolysed black mass, hydrometallurgical route	Pilot	50 kg/hr	S8
RecycLiCo/Kemetco, Vancouver	Demonstration	500 kg/day design (815 kg/day achieved) — cathode waste input, downstream of mechanical black-mass production	S9

Supplemental references

S1. Mining See (2026). Black mass recycling boom: How Fortum, Umicore, Altilium and Nth Cycle are turning Europe's battery waste into critical raw materials. https://www.miningsee.eu/black-mass-recycling-boom-how-fortum-umicore-altilium-and-nth-cycle-are-turning-europes-battery-waste-into-critical-raw-materials/ (accessed 3 August 2026).

S2. Circular Economy & Battery Analyst (2026). EV battery recycling 2026: Complete process guide, top companies, costs & regulations. https://energy-solutions.co/articles/sub/ev-battery-recycling-what-happens (accessed 3 August 2026).

S3. BASF (2025). BASF starts commercial operation of black mass plant for battery recycling in Schwarzheide, Germany. https://www.basf.com/global/en/media/news-releases/2025/06/p-25-112 (accessed 3 August 2026).

S4. Lazzaro, N. (2022). Li-cycle targets over 6,500 mt black mass output in 2022. https://www.spglobal.com/energy/en/news-research/latest-news/energy-transition/031722-li-cycle-targets-over-6500-mt-black-mass-output-in-2022 (accessed 3 August 2026).

S5. Fortunebusinessinsights (2026). Black mass recycling market size, share & industry analysis, by feedstock type (production & process scrap and end of life material), by chemistry (nickel-based (NMC & NCA), lithium iron phosphate (LFP), and others), by recovered materials (lithium (Li), nickel (Ni), cobalt (Co), copper (Cu), manganese (Mn), graphite, and others) and regional forecast, 2026-2034. https://www.fortunebusinessinsights.com/black-mass-recycling-market-108661 (accessed 3 August 2026).

S6. Spector, J. (2024). Fire, delays, and financial woes: Battery recycling had a rough 2024. https://www.canarymedia.com/articles/recycling-renewables/ev-battery-recycling-had-a-rough-2024 (accessed 3 August 2026).

S7. Blois, M. (2022). Umicore wants to build the world's largest battery recycling plant. https://cen.acs.org/environment/recycling/Umicore-wants-build-worlds-largest/100/i23 (accessed 3 August 2026).

S8. Woeste, R., Drude, E.-S., Vrucak, D., Klöckner, K., Rombach, E., Letmathe, P., and Friedrich, B. (2024). A techno-economic assessment of two recycling processes for black mass from end-of-life lithium-ion batteries. Applied Energy *361*, 122921. https://doi.org/10.1016/j.apenergy.2024.122921.

S9. RecycLiCo (2021). RecycLiCo battery materials advances demonstration plant for lithium-ion battery recycling and upcycling project. https://recyclico.com/american-manganese-advances-demonstration-plant-for-lithium-ion-battery-recycling-and-upcycling-project/ (accessed 3 August 2026).
